# Therapeutic gene correction of *HBB* frameshift CD41-42 (-TCTT) deletion in human hematopoietic stem cells

**DOI:** 10.1007/s44307-024-00053-5

**Published:** 2025-01-02

**Authors:** Qianyi Liu, Xinyu Li, Hui Xu, Ying Luo, Lin Cheng, Junbin Liang, Yuelin He, Haiying Liu, Jianpei Fang, Junjiu Huang

**Affiliations:** 1 MOE Key Laboratory of Gene Function and Regulation, State Key Laboratory of Biocontrol, School of Life Sciences, Sun Yat-Sen University, Guangzhou, 510275 Guangdong China; 2 Department of Pediatrics, Sun Yat-Sen Memorial Hospital, Sun Yat-Sen University, No.107, West Yan Jiang Road, Guangzhou, 510120 Guangdong China; 3 Reforgene Medicine, Guangzhou, 510535 Guangdong China; 4 Dongguan Taixin Hospital, Dongguan, 523170 Guangdong China; 5 Key Laboratory of Reproductive Medicine of Guangdong Province, the, First Affiliated Hospital and School of Life Sciences , Sun Yat-Sen University, Guangzhou, 510275 Guangdong China

**Keywords:** Gene editing therapy, β-thalassemia, *HBB* CD41-42 (-TCTT), CRISPR/Cas9, Hematopoietic stem and progenitor cells

## Abstract

Β-thalassemia is one of the global health burdens. The CD41-42 (-TCTT) mutation at *HBB* is the most prevalent pathogenic mutation of β-thalassemia in both China and Southeast Asia. Previous studies focused on repairing the *HBB* CD41-42 (-TCTT) mutation in β-thalassemia patient-specific induced pluripotent stem cells, which were subsequently differentiated into hematopoietic stem and progenitor cells (HSPCs) for transplantation. In this study, we directly applied the CRISPR/Cas9-based gene editing therapy to correct the *HBB* CD41-42 (-TCTT) mutation in patient-derived HSPCs. The effective editing induced by Cas9:sgRNA ribonucleoprotein and single-stranded oligodeoxynucleotides (ssODNs) was confirmed in HUDEP-2 cell lines harboring the *HBB* CD41-42 (-TCTT) mutation. Further correction of heterozygote and homozygote *HBB* CD41-42 (-TCTT) mutations in patient-derived HSPCs resulted in a 13.4–40.8% increase in the proportion of HBB-expressing (HBB +) cells following erythroid differentiation in vitro. At 16 weeks post-xenotransplantation of the edited HSPCs into coisogenic immunodeficient mice, the reparation efficiency in engrafted bone marrow was 17.21% ± 3.66%. Multiparameter flow cytometric analysis of the engrafted bone marrow showed an increase in the percentage of HBB + cells without impairing the ability of engraftment, self-renewal, and multilineage hematopoietic repopulation of HSPCs. For the safety evaluation, 103 potential off-target sites were predicted by SITE-seq and CRISPOR, with one site displaying significant off-target editing. Since this off-target site is located in the intergenic region, it is presumed to pose minimal risk. Taken together, our study provides critical preclinical data supporting the safety and efficacy of the gene therapy approach for *HBB* CD41-42 (-TCTT) mutation.

**Supplementary Information:**

The online version contains supplementary material available at 10.1007/s44307-024-00053-5.

## Introduction

Β-thalassemia, one of the most prevalent monogenic diseases worldwide, is caused by the mutations or deletions in the β-globin gene (*HBB*), leading to an imbalance between α-globin and β-globin chains, resulting in hemolysis and ineffective erythropoiesis (Taher et al. 2021). Patients with β-thalassemia major experience numerous complications affecting the heart, liver, spleen, endocrine glands, and other organs (Galanello & Origa 2010; Taher et al. 2018), and rely on regular blood transfusions to sustain life (Langer 1993).

To date, over 500 β-thalassemia mutations have been identified (Kountouris et al. 2014). Among them, the CD41-42 (-TCTT) mutation in *HBB* is the most common pathogenic variant in populations from China and Southeast Asia (Cao & Kan 2013; Huang et al. 1990; Lai et al. 2017; Zhang et al. 2008). In China, the gene frequency of the CD41-42 (-TCTT) mutation reaches approximately 31.8% among individuals with β-thalassemia (Huang et al. 1990). This 4-bp deletion results in a frameshift and introduces an early stop codon at the new 59th codon, causing premature truncation of β-globin synthesis (Kimura et al. 1983). Patients with the homozygotes CD41-42 (-TCTT) mutation, or compound heterozygotes carrying CD41-42 (-TCTT) mutation alongside other β^0^ or β^+^-thalassemia mutations, typically present with β-thalassemia major and require regular transfusion to survive (Laosombat, Wongchanchailert, Sattayasevana, Wiriyasateinkul, & Fucharoen, 2001).

Hematopoietic stem cell transplantation (HSCT) remains the primary curative option for β-thalassemia major, yet it is challenged by the limited availability of HLA-matched donors and graft-related complications (Thompson et al. 2018). Zynteglo (Bluebird), involving the addition of a modified β-globin gene (*HBB* ^*T87Q*^) followed by autologous HSCT, has been approved by both the EMA and FDA for treating transfusion-dependent β-thalassemia, though concerns persist regarding the risk of insertional oncogenesis (F. Locatelli et al. 2022a, b; Magrin et al. 2022). Gene editing therapy has emerged as a promising alternative. The clustered regularly interspaced short palindromic repeats (CRISPR)/CRISPR-associated protein 9 (Cas9) nuclease system, widely applied in gene therapy (H. Frangoul et al. 2021; Gillmore et al. 2021; Wei et al. 2019; Wen et al. 2022; Zittersteijn et al. 2020), was guided by single-guide RNA (sgRNA) to generate double-strand break at the desired site, activating either the nonhomologous end-joining (NHEJ) or homology-directed repair (HDR) pathways (Cong et al. 2013; Jinek et al. 2012). NHEJ, active throughout the cell cycle, often results in imprecise small insertions or deletions (indels) formation (Lieber 2008), while HDR, normally occurring in late S and G2 phases, enables precise and predictable gene editing (Kass & Jasin 2010; Moynahan & Jasin 2010). Current NHEJ-based therapies for β-thalassemia primarily target the disruption of BCL11A interaction with the γ-globin promoter, thereby reactivating fetal hemoglobin (HbF) expression (De Dreuzy et al. 2019; Haydar Frangoul et al. 2022; Fu et al. 2022; R. Liu et al. 2022; Franco Locatelli et al. 2022a, b; Smith et al. 2019). This approach mimics the hereditary persistence of fetal hemoglobin (HPFH) to alleviate anemia (Canver & Orkin 2016; Wienert et al. 2018). The related cell-based gene therapy, Casgevy (Co-developed by Vertex Pharmaceuticals and CRISPR Therapeutics) has received approval in the United Kingdom and the United States. However, differences in oxygen affinity between HbF and normal adult hemoglobin (HbA) may limit HbF's capacity to fully substitute HbA in oxygen transport (Adachi et al. 1997).

Precise and traceless repair of the *HBB* gene via HDR presents a promising therapeutic strategy for β-thalassemia. Several studies have attempted to correct the *HBB* CD41-42 (-TCTT) mutation in β-thalassemia patient-specific induced pluripotent stem cells (iPSCs) using single-strand oligodeoxynucleotides (ssODNs) as DNA donors along with the CRISPR/Cas9 system, achieving up to 54% biallelic correction and 25.5% monoallelic correction following GFP-positive cells sorting (Y. Liu et al. 2017; Niu et al. 2016; Xian et al. 2020; Y. Yang et al. 2016). However, the potential tumorigenicity of iPSCs and the challenges of hematopoietic differentiation limit the application of iPSC-based gene therapy (Han et al. 2021; Papapetrou 2017). In contrast, using the HSCT platform, it is safer and more practical to correct patient-derived hematopoietic stem and progenitor cells (HSPCs), followed by autologous HSCT (Canver & Orkin 2016; Haydar Frangoul et al. 2022; Fu et al. 2022; Franco Locatelli et al. 2022a, b). Therefore, we aim to develop a CRISPR/Cas9-based gene editing therapy directly targeting the *HBB* CD41-42 (-TCTT) mutation in patient-derived HSPCs.

Here, we evaluated the effectiveness of CRISPR/Cas9-based gene therapy in correcting the CD41-42 (-TCTT) mutation in heterozygotes and homozygotes β-thalassemia patient-derived HSPCs. The repopulation potential and therapeutic effects of the edited HSPCs were analyzed via multiparameter flow cytometry following the transplantation into NCG-X mice. This work aims to develop a novel CRISPR/Cas9-based gene editing strategy for treating β-thalassemia with the CD41-42 (-TCTT) mutation in patient-derived HSPCs, providing preclinical in vitro and in vivo evidence for further translation into clinical gene therapy.

## Patients and methods

### Patient-derived HSPCs harvesting

The human CD34^+^ HSPCs from the mobilized peripheral blood of patients with the *HBB* CD41-42 (-TCTT) mutation were collected via apheresis at Sun Yat-sen Memorial Hospital, with approval from the institution’s Ethical Committee (No. 2020–328). All patients provided informed consent. The CD34^+^ HSPCs were purified using FcR Blocking Reagent (Miltenyi) and CD34 MicroBeads UltraPure (Miltenyi) according to the manufacturer’s instructions, followed by cryopreservation.

### Cell culture and transfection

The HUDEP-2 cells (Kurita et al. 2013) were cultured in StemSpan Serum-Free Expansion Medium (SFEM, Stemcell Technologies) supplemented with 1% Penicillin–Streptomycin solution (10,000 U/mL stock), 50 ng/mL recombinant human stem cell factor (SCF, Peprotech), 3 IU/mL Epoetin alfa (Epogen, Amgen), 0.4 μg/mL dexamethasone, and 1 μg/mL doxycycline. Transfection of the HUDEP-2 cells was performed via electroporation (Lonza, 4D X-unit, FF-120). To generate the HUDEP-2-CD41-42 M cell line, the RNP complexes containing 15 μg SpCas9 and 250 pmol sgRNA_WT, along with 100 pmol ssODNs_CD41-42 M were delivered through electroporation. The transfected HUDEP-2 cells were diluted to one cell per 0.1 mL of medium and cultured in 96-well plates to isolate individual clones. For gene correction in HUDEP-2-CD41-42 M, the RNP complexes containing 15 μg SpCas9 and 250 pmol sgRNA_1 or sgRNA_2, as well as 100 pmol ssODNs were similarly delivered via electroporation.

For gene correction, the CD34^+^ HSPCs were thawed on Day 0 and cultured in Serum-free Stem Cell Growth Medium (SCGM, CellGenix) supplemented with 100 ng/mL Stem Cell Factor (SCF, Peprotech), 100 ng/mL human thrombopoietin (TPO, Peprotech), and 100 ng/mL recombinant human Flt3-ligand (Flt3-L, Peprotech). 48 h post-thawing, the HSPCs were electroporated using Lonza 4D Nucleofector (V4XP-3032, Lonza 4D X-unit, EO-100) following the manufacturer’s instructions. RNP complexes, containing 15 μg SpCas9 and 250 pmol corresponding sgRNAs, along with 100 pmol ssODNs were transfected into 4 × 10^5^ HSPCs.

The SpCas9 protein used in this study was purchased from KACTUS. All the sgRNAs were chemically modified with 2’-O-methyl and 3’-phosphorothioate groups at the first three 5’ and 3’ terminal RNA residues (GenScript). The ssODNs were chemically modified with phosphorothioate groups at the first two 5’ and 3’ terminal DNA residues (GenScript). The detailed sequences of sgRNAs and ssODNs are listed in Table S1.

### Erythroid differentiation of CD34^+^ HSPCs *in vitro*

For erythroid differentiation of CD34^+^ HSPCs in vitro, the electroporated cells were transferred to StemSpan SFEM II with StemSpan Erythroid Expansion supplement (STEMCELL) and cultured for 21 days, maintaining an optimal cell density (< 10^6^ cells/mL). The medium was replaced every 3–4 days. During the first 13 days of erythroid differentiation, cell counts were performed using acridine orange and propidium iodide (AO/PI, Countstar) staining to monitor the erythroid differentiation.

### Transplantation of CD34 + HSPCs into mice

All animal experiments were approved by the Institutional Animal Care and Use Committee of Sun Yat-sen University (SYSU-IACUC-2020-B079). The non-obese diabetic (NOD)/ShiLtJGpt-Prkdc^em26Cd52^Il2rg^em26Cd22^kit^em1Cin(V831M)^/GptCrl coisogenic immunodeficient mice (NCG-X) were purchased and housed in GemPharmatech. For xenotransplantation, the HSPCs were frozen 16 h post-electroporated until transplantation. Each non-irradiated NCG-X female mouse (4–6 weeks old) received an intravenous injection of 4 × 10^5^ edited or untransfected HSPCs derived from β-thalassemia patients with the homozygote *HBB* CD41-42 (-TCTT) mutation.

The peripheral blood and bone marrow samples were collected at the indicated time, mainly at 16 weeks or 26 weeks post-transplantation. For flow cytometry analysis, cells were incubated with Human TruStain FcX (BioLegend, 422,302) and TruStain fcX (anti-mouse CD16/32, BioLegend, 101,320) blocking antibodies, then stained with the following antibody to meet different needs: anti-mouse CD45 antibody APC (30-F11, Biolegend, 103,112), anti-mouse CD45 antibody PE-CY7 (30-F11, BDeBioscience, 552,848), anti-human CD45 antibody PE (2D1, Biolegend, 368,512), anti-human CD45 antibody APC-CY7 (2D1, Biolegend, 368,516), anti-human CD45 antibody Brilliant Violet 510™ (2D1, Biolegend, 368,525), anti-human CD34 antibody PerCP/Cyanine5.5 (561, Biolegend, 343,612), anti-human CD34 antibody PE (561, Biolegend, 343,602), anti-human CD33 antibody APC (WM53, Biolegend, 303,408), anti-human CD3 antibody APC (UCHT1, Biolegend, 300,412), anti-human CD19 antibody FITC (HIB19, Biolegend, 302,206), anti-human CD56 antibody PE (5.1H11, Biolegend, 362,508), anti-human CD16 antibody FITC (3G8, Biolegend, 302,006), anti-human CD235a antibody FITC (H1264, Biolegend, 349,104), anti-human CD235a antibody APC (H1264, Biolegend, 349,113), and Hemoglobin β antibody (Santa Cruz, sc-21757). Antibodies were diluted according to the manufacturer's instructions.

The human engraftment was evaluated as hCD45 + cells/(hCD45 + cells + mCD45 + cells) × 100%. The percentages of HSPCs (hCD45 + /hCD34 +), T cells (hCD45 + /hCD3 +), B cells (hCD45 + /hCD19 +), and myeloid cells (hCD45 + /hCD33 +) were analyzed within the hCD45 + cell population. The percentage of NK cells (hCD45 + /hCD56 + /hCD16 +) was assessed within the hCD45 + /hCD56 + cell population. The percentage of erythroid cells (hCD235a +) was assessed in the total cell population, while HBB-expressing cells (HBB +) were evaluated within the hCD235a + population. All flow cytometry analyses were conducted using FlowJo v.10.

### Evaluation of editing efficiency

The editing efficiency was assessed 96 h after electroporation, and at 16 weeks or 26 weeks after xenotransplantation. Genomic DNA was extracted and amplified using the primers listed in Table S1 for Sanger Sequencing or next-generation sequencing (NGS, Novaseq 6000). The Sanger Sequencing data was analyzed by ICE, while the NGS data was analyzed by CRISPResso (Clement et al. 2019).

### Assessment of *HBB* expression in mRNA levels and protein levels

Progeny cells from edited HSPCs, after 21-day erythroid differentiation in vitro, were lysed in RNAiso (TAKARA) for total RNA extraction. The expression of *HBB* mRNA was detected using the specific primers targeting the corrected β-globin transcript (listed in Table S1). GADPH served as an endogenous control for relative *HBB* expression, with the Edited group normalized to the Ctrl group. Mean expression levels were calculated from three technical replicates. HBB protein levels were analyzed by western blot as previously reported (Cosenza et al. 2021), using the following antibodies: HBA (Santa Cruz, sc-514378), HBB (Santa Cruz, sc-21757), and β-actin (Cell Signaling Technology, 3700S).

### Prediction and assessment of candidate off-target sites.

The selective enrichment and identification of tagged genomic DNA ends by sequencing (SITE-seq) was performed as previously described (Cameron et al. 2017). The purified genome was treated with either 90 nmol or 256 nmol Cas9:sgRNA_1 RNP for DNA cleavage in vitro, and the breakpoint sites with more than 5 reads detected were considered the candidate off-target sites. The CRISPOR program was also used to predict candidate off-target sites with fewer than 4 mismatches. In total, 103 candidate off-target sites were predicted for further analysis, identified by both SITE-seq with 90 nmol RNP and either SITE-seq with 256 nmol RNP or CRISPOR.

The potential off-target sites were all assessed in untransfected HSPCs, edited HSPCs, and cells from the engrafted bone marrow of mice, using sample-specific barcodes and adapters for amplicon sequencing (Novaseq 6000). The frequency of indels at these off-target sites was analyzed by CRISPResso (Clement et al. 2019). Sanger sequencing was performed to further confirm the off-target activity at OT001.

### Statistical analysis

The data are expressed as the mean ± standard deviation (SD). Two-tailed unpaired *t*-test (GraphPad 8.0.2) were used to analyze the statistical significance between the edited group and the control. The significance was shown by *p < 0.05; **p < 0.01; or ***p < 0.001.

## Results

### Screening for sgRNAs targeting *HBB* CD41-42 (-TCTT) mutation in the disease modeling cell lines

**Fig. 1:**
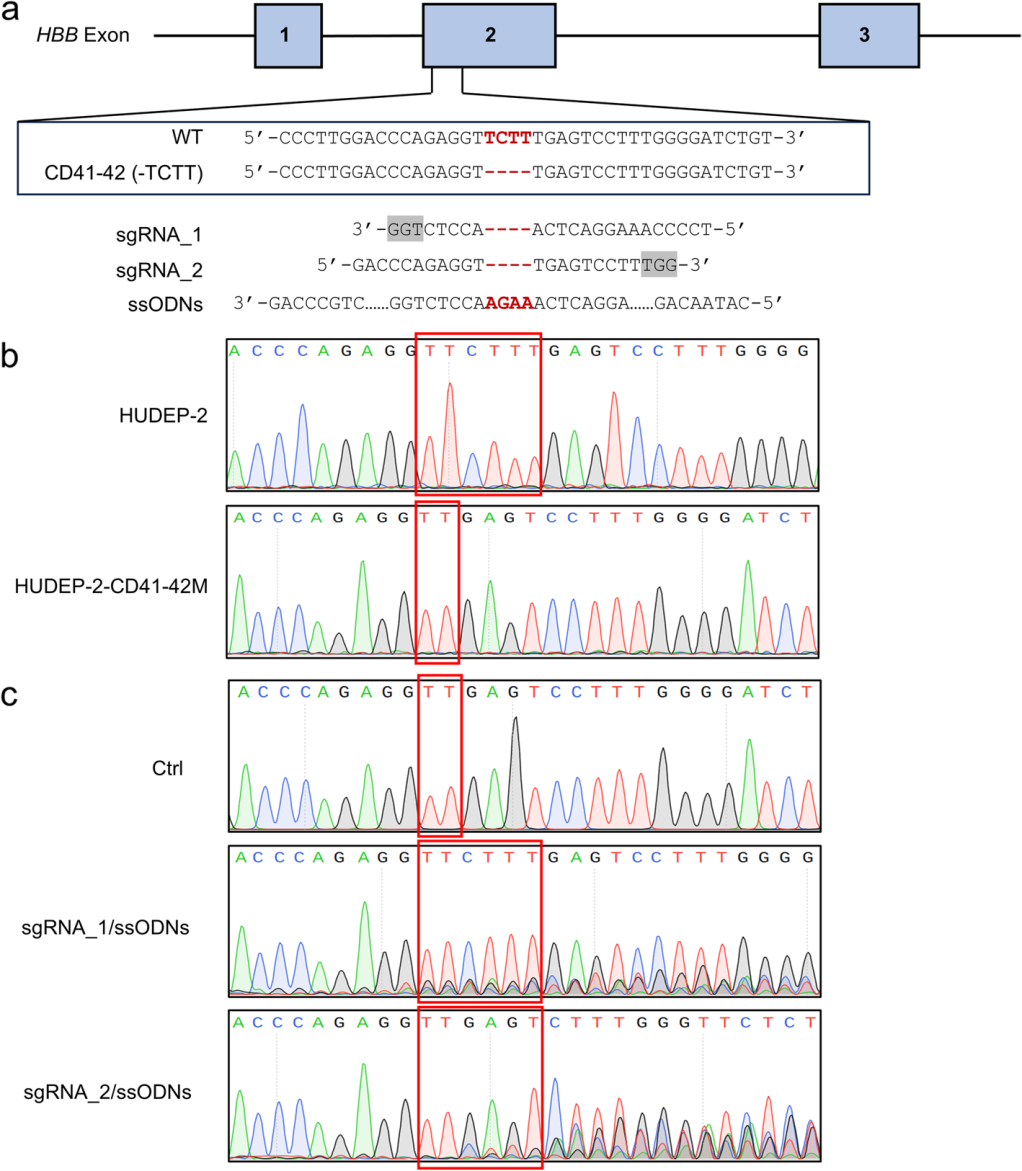
Correcting *HBB* CD41-42 (-TCTT) mutation in the disease modeling cell lines. **a** Schematic of *HBB* CD41-42 (-TCTT) mutation. The exons of *HBB* were labeled with blue boxes. The *HBB* CD41-42 (-TCTT) mutation was indicated in red. The sequences of sgRNAs for gene correction were shown in the middle with the PAM sequence shown in a grey background. The sequences of ssODNs for gene correction are shown below. **b** Sanger sequencing chromatographs of the PCR amplicons of the wild-type HUDEP-2 and the *HBB* CD41-42 (-TCTT) mutant stable HUDEP-2 cell lines (HUDEP-2-CD41-42 M), with the homozygotes 4 bp loss at the *HBB* CD41-42 (-TCTT) mutation site. **c** Representative Sanger sequencing chromatographs showing the correction efficiency of *HBB* CD41-42 (-TCTT) mutation in the HUDEP-2-CD41-42 M cell line mediated by sgRNA_1 or sgRNA_2 with ssODNs as the template for reparation. The *HBB* CD41-42 (-TCTT) mutation was labeled with red boxes

For precise and effective gene correcting of *HBB* CD41-42 (-TCTT) mutation, we adopted an HDR-based gene therapy strategy using the electroporation of SpCas9:sgRNA ribonucleoprotein (RNP) and ssODNs as donors. Two sgRNAs targeting the *HBB* CD41-42 (-TCTT) mutation and meeting the NGG PAM requirement were designed. Meanwhile, the ssODNs were synthesized as repair templates (Fig. 1a).

The stable HUDEP-2 erythroid cell line with homozygotes CD41-42 (-TCTT) mutations (HUDEP-2-CD41-42 M) was generated by electroporating SpCas9:sgRNA_WT RNP and ssODNs_CD41-42 M, facilitating the screening of different sgRNAs (Kurita et al. 2013) (Fig. S1a). The 4-bp deletion in the β-globin coding sequence of the HUDEP-2 cell clone was confirmed through both Sanger sequencing and deep sequencing (Fig. 1b and Fig. S1b).

We subsequently assessed the editing efficiency of sgRNAs in the HUDEP-2-CD41-42 M cell line. At 96 h post-electroporation, we extracted the cell genome and amplified the target site for sequencing. Whether the 4-bp deletion was repaired with TCTT or TTTT, the wild-type amino acid sequence of β-globin was restored, indicating successful functional reparation. Cells transfected with sgRNA_1/ssODNs showed higher gene correction efficiency compared to those transfected with sgRNA_2/ssODNs (Fig. 1c and Fig. S1c,d). To our surprise, HUDEP-2 transfected with sgRNAs alone, without ssODN templates, also achieved accurate and effective gene correction, though with a slight reduction in editing efficiency–5.89% and 3.62% less compared to those with ssODNs (Fig. S1c,d). This unexpectedly high efficiency in gene correction might be attributed to the paralog gene *HBD* serving as an internal template, as observed in human tripronuclear zygotes and HSPCs (Liang et al. 2015; F. Yang et al. 2023).

Collectively, we successfully corrected the *HBB* CD41-42 (-TCTT) mutation in the HUDEP-2-CD41-42 M cell line. The combination of Cas9:sgRNA_1 RNP and ssODNs demonstrated the highest efficiency in precise editing, making it a promising approach for further genetic correction in HSPCs.

### Therapeutic gene correction of the *HBB* CD41-42 (-TCTT) mutation in patient-derived HSPCs

**Fig. 2:**
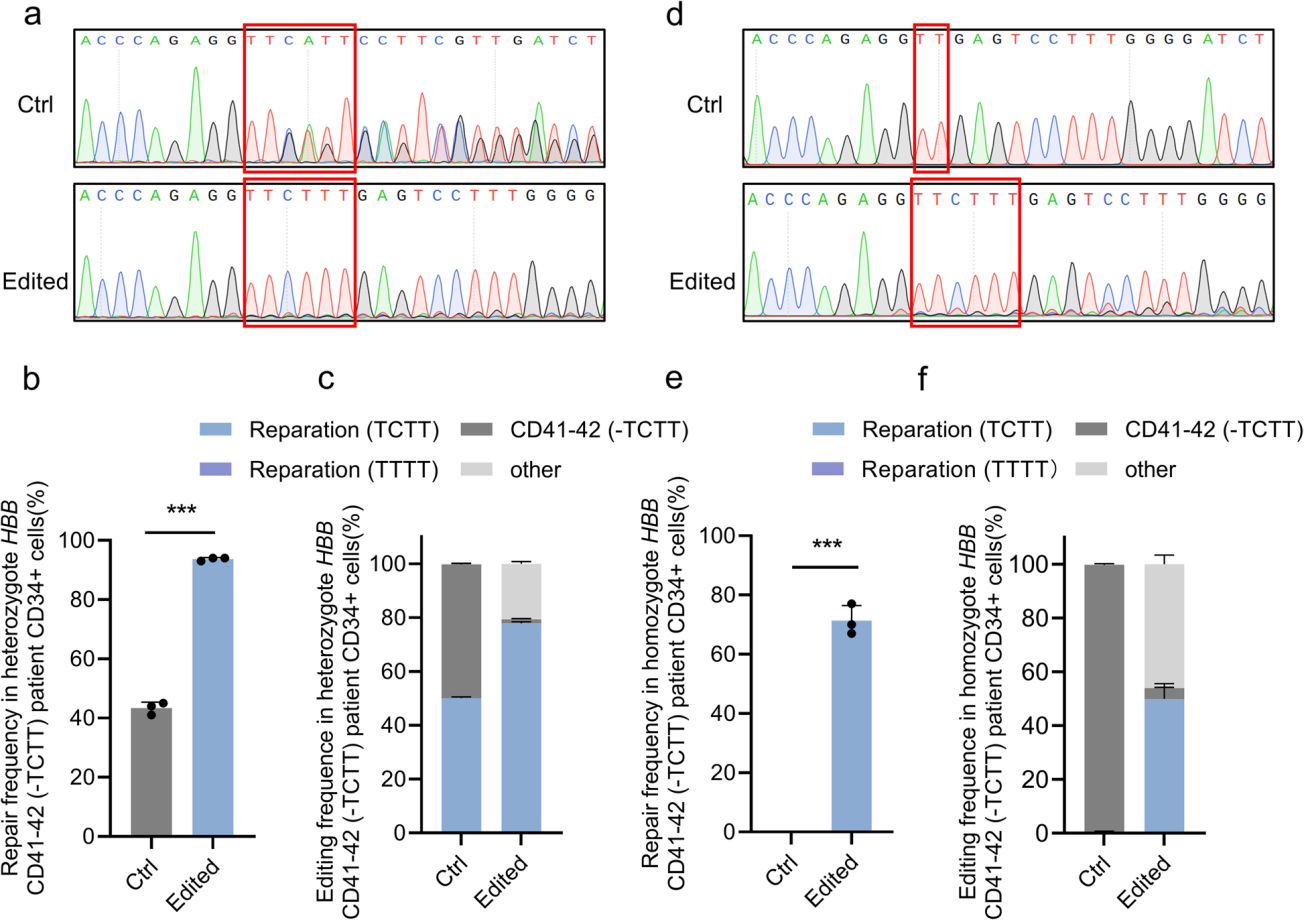
Correcting efficiency in heterozygote or homozygote *HBB* CD41-42 (-TCTT) patient-derived CD34^+^ cells**. a** Representative Sanger sequencing chromatographs showing the effective gene reparation in heterozygote *HBB* CD41-42 (-TCTT) patient-derived CD34^+^ cells. The red boxes indicated the *HBB* CD41-42 (-TCTT) mutation sites. (**b**) Statistics of functional reparation frequency of *HBB* CD41-42 (-TCTT) mutation in heterozygote *HBB* CD41-42 (-TCTT) patient-derived CD34^+^ cells detected by Sanger sequencing and calculated by ICE analysis. (**c**) Statistics of particular reparation frequency of *HBB* CD41-42 (-TCTT) mutation in heterozygote *HBB* CD41-42 (-TCTT) patient-derived CD34^+^ cells detected by NGS. **d** Representative Sanger sequencing chromatographs showing the effective gene reparation in homozygote *HBB* CD41-42 (-TCTT) patient-derived CD34^+^ cells. The red boxes indicated the *HBB* CD41-42 (-TCTT) mutation sites. **e** Statistics of functional reparation frequency of *HBB* CD41-42 (-TCTT) mutation in homozygote *HBB* CD41-42 (-TCTT) patient-derived CD34^+^ cells detected by Sanger sequencing and calculated by ICE analysis. **f** Statistics of particular reparation frequency of *HBB* CD41-42 (-TCTT) mutation in homozygote *HBB* CD41-42 (-TCTT) patient-derived CD34.^+^ cells detected by NGS. In panel a-f, Ctrl indicated the untransfected HSPCs, while Edited indicated the HSPCs transfected with Cas9:sgRNA_1 RNP and ssODNs as templates. In panels b-c and e–f, the data represent the mean ± SD for 3 replicates using HSPCs from three independent donors. ***, *P* < 0.001 (by unpaired *t*-test)

To evaluate the potential therapeutic benefits of this CRISPR/Cas9-based gene correction strategy in clinically relevant models, we targeted the *HBB* CD41-42 (-TCTT) mutation in patient-derived HSPCs. Cells derived from three different donors carrying heterozygote *HBB* CD41-42 (-TCTT) mutation were effectively edited using the Cas9:sgRNA_1 RNP and ssODNs, as confirmed by Sanger sequencing (Fig. 2a,b). The nonpathogenic allele frequency, including alleles with TCTT or TTTT reparations, increased from 50.13% ± 0.46% to 77.93% ± 0.53% after gene correction (Fig. 2c and Fig. S2a). Encouraged by this, we applied the same approach to homozygote *HBB* CD41-42 (-TCTT) mutation HSPCs derived from 3 different patients, achieving successful gene editing (Fig. 2d,e). The gene correction was confirmed by NGS, with 49.92% ± 4.31% functional gene reparation (Fig. 2f and Fig. S2b).

**Fig. 3:**
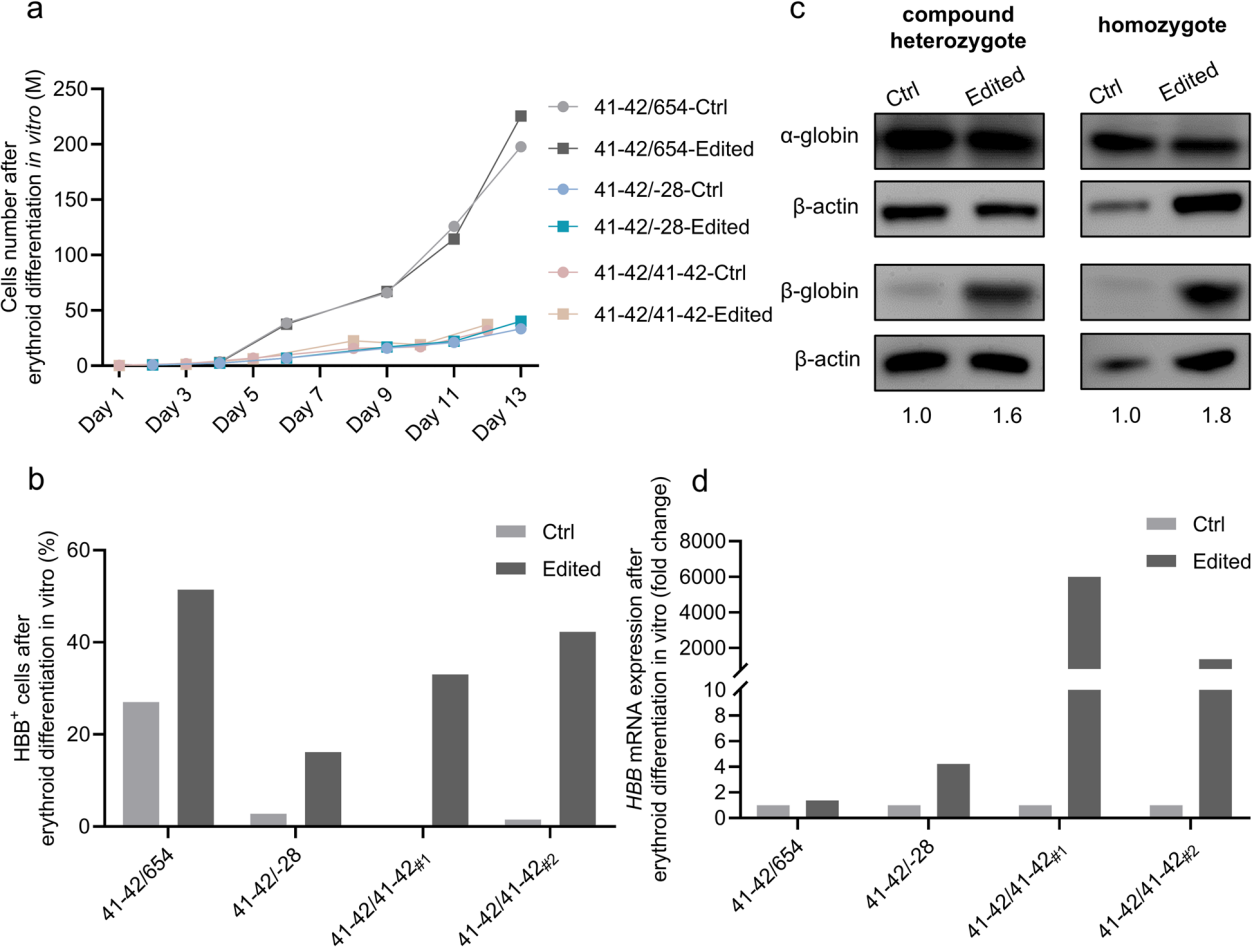
Correcting *HBB* CD41-42 (-TCTT) mutation in patient-derived CD34 + cells induced HBB expression. **a** The growth curve analysis of the unedited or edited heterozygote or homozygote *HBB* CD41-42 (-TCTT) patient-derived CD34 + cells during erythroid differentiation. The progeny cells were counted after AO/PI staining. Samples labeled with the same genotype derived from the same donor. **b** The percentage of HBB + cells after erythroid differentiation (21 days) of the unedited or edited HSPCs, which is detected by flow cytometry after cell immunostaining. Samples were derived from two independent donors with heterozygote *HBB* CD41-42 (-TCTT) mutation and two independent donors with homozygote *HBB* CD41-42 (-TCTT) mutation. **c** The expression of α-globin and β-globin after erythroid differentiation (21 days) of the unedited or edited heterozygote (41–42/654) or homozygote (41–42/41–42_#2_) *HBB* CD41-42 (-TCTT) patient-derived CD34 + cells. β-actin served as an internal control to determine the relative expression of α-globin and β-globin. The β-globin/α-globin ratio normalized to the control is shown below. The full uncropped blots images were given in Online Resource. **d** HBB mRNA expression quantified by RT-qPCR after erythroid differentiation (21 days) of the unedited or edited heterozygote or homozygote *HBB* CD41-42 (-TCTT) patient-derived CD34 + cells. GADPH mRNA expression was used as input control. The HBB mRNA expression in the Edited group was normalized to that in the Ctrl group. Samples were derived from two independent donors with heterozygote *HBB* CD41-42 (-TCTT) mutation and two independent donors with homozygote *HBB* CD41-42 (-TCTT) mutation, which is consistent with the samples shown in panel b. Ctrl indicated the untransfected HSPCs, while Edited indicated the HSPCs transfected with Cas9:sgRNA_1 RNP and ssODNs as templates

We subsequently induced erythroid differentiation of edited CD34 + HSPCs in vitro. Using AO/PI staining and cell counting, we quantified the progeny cells during erythroid differentiation before the initiation of enucleation of cultured erythroid cells. The results demonstrated that the edited HSPCs exhibited comparable growth curves to the control group throughout erythroid differentiation, regardless of whether the HSPCs carried homozygote or heterozygote *HBB* CD41-42 (-TCTT) mutations (Fig. 3a). This finding suggested that the CRISPR/Cas9-mediated gene correction did not impair the proliferation or erythroid differentiation potential of HSPCs.

After 21 days of erythroid differentiation, we assessed the therapeutic impact of the gene correction targeting the homozygote or heterozygote *HBB* CD41-42 (-TCTT) mutation in late-stage erythroblasts. The proportion of HBB + cells was evaluated by flow cytometry, revealing a 13.4–40.8% increase in cells derived from edited HSPCs compared to the unedited cells (Fig. 3b and Fig. S3). Additionally, the enhanced β-globin/α-globin ratio following gene correction was validated by western blot analysis (Fig. 3c). The results of quantitative reverse transcription PCR demonstrated that the corrected β-globin transcript levels increased by 1.39–4.23 fold in cells with the heterozygote *HBB* CD41-42 (-TCTT) mutation, and by 1368.09–5990.01 fold in cells with the homozygote mutation (Fig. 3d). This discrepancy is likely attributable to baseline *HBB* expression in heterozygote mutation cells and the heterogeneity between different donors' HSPCs.

In summary, patient-derived HSPCs with heterozygote or homozygote *HBB* CD41-42 (-TCTT) mutation could be functionally repaired through Cas9-mediated HDR editing, without impairing their erythroid differentiation potential.

### Correction of the *HBB* CD41-42 (-TCTT) mutation in long-term repopulating HSCs recovered *HBB* expression in erythroid progeny *in vivo*

**Fig. 4:**
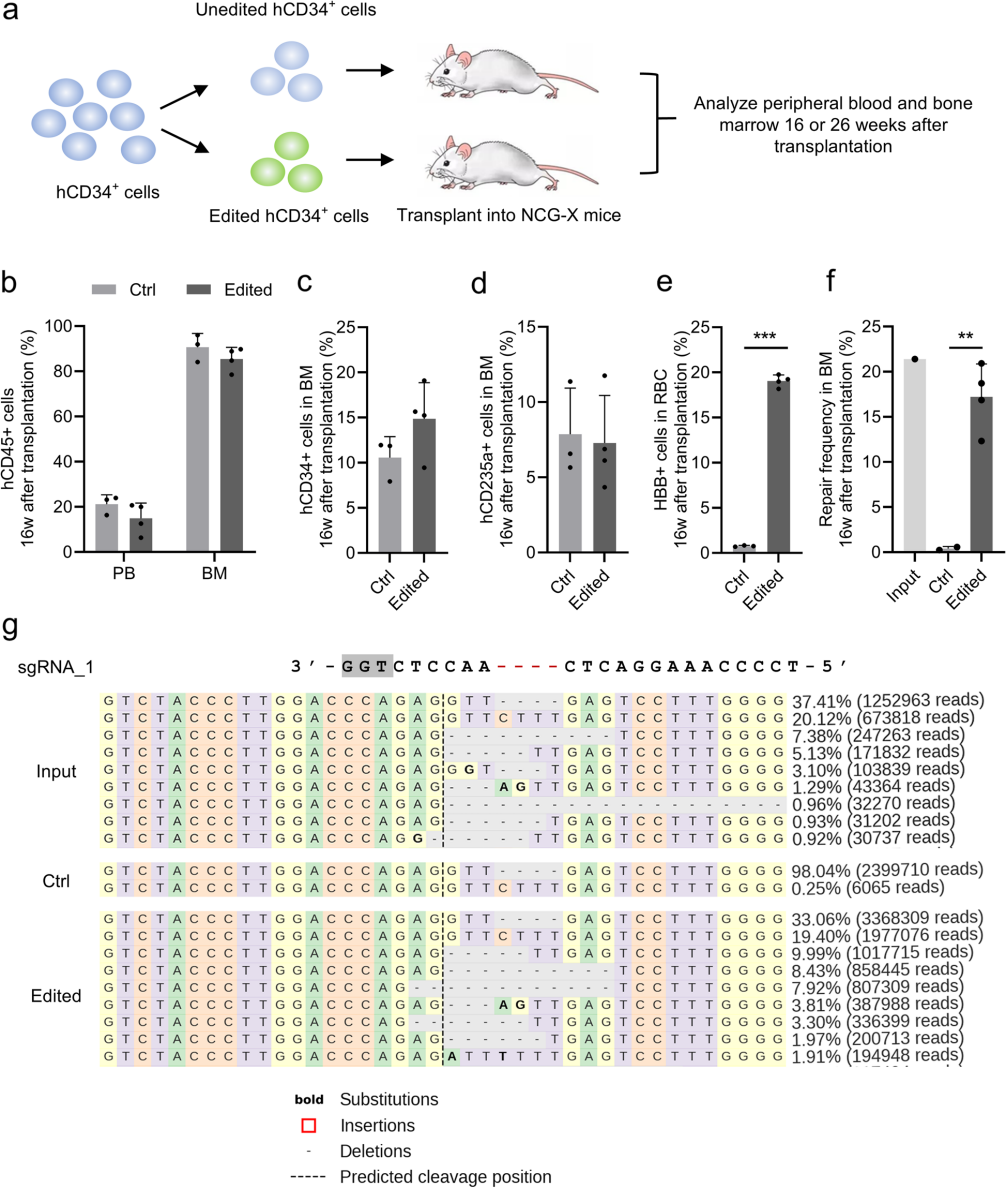
Xenotransplantation of patient-derived gene-edited CD34.^+^ cells into NCG-X mice improved the *HBB* expression. **a** Schematic of the xenotransplantation of patient-derived CD34 + cells into NCG-X mice. An equal number of unedited (Ctrl) or edited hCD34 + cells transfected with Cas9:sgRNA_1 RNP and ssODNs as templates were transplanted into NCG-X mice, which supported human erythroid reconstitution without irradiation. The ability of human engraftment, self-renewal, erythroid differentiation, and the therapeutic benefit in improved *HBB* expression were analyzed 16 or 26 weeks after transplantation. **b** The human engraftment in peripheral blood (PB) or bone marrow (BM) of engrafted mice 16 weeks after transplantation, shown as the percentage of human (h) CD45 + cells within the population of CD45 + cells (mouse and human) detected by flow cytometry. **c** The self-renewal of HSPCs in the bone marrow of engrafted mice 16 weeks after transplantation, shown as the percentage of hCD34 + cells within the population of hCD45 + cells detected by flow cytometry. **d** The erythroid differentiation of HSPCs in the bone marrow of engrafted mice 16 weeks after transplantation, shown as the percentage of hCD235a + cells detected by flow cytometry. **e** The percentage of HBB + cells within the population of hCD235a + cells in the bone marrow of engrafted mice 16 weeks after transplantation, which was detected by flow cytometry. **f** Statistics of the functional reparation frequency of *HBB* CD41-42 (-TCTT) mutation in the input HSPCs and the cells derived from the bone marrow of engrafted mice 16 weeks after transplantation, which was detected by NGS. **g** Representative DNA profiles of the input HSPCs and the cells derived from the bone marrow of engrafted mice 16 weeks after transplantation, which was detected by NGS. The sequence of sgRNA_1 was shown at the top with the PAM sequence shown in grey background and the *HBB* CD41-42 (-TCTT) mutation shown in red. The bordered sequences indicated the substitutions. The red boxes indicated the insertions. The short line indicated the deletions. The vertical dotted line indicated the predicted cleavage position. In panel b-g, Ctrl indicated the mice transplanted with untransfected HSPCs, while Edited indicated the mice transplanted with HSPCs transfected with Cas9:sgRNA_1 RNP and ssODNs as templates. In panel b-f, the data represent the mean ± SD for 2 to 4 engrafted mice transplanted by unedited or edited homozygote *HBB* CD41-42 (-TCTT) patient-derived CD34 + cells. Each dot represented a single recipient mouse. ***, *P* < 0.001, **, *P* < 0.01 (by unpaired *t* test)

To further assess the functional potential of the corrected HSPCs, an equal number of unedited and edited HSPCs derived from patients with homozygous *HBB* CD41-42 (-TCTT) mutations were transplanted into the non-obese diabetic (NOD)/ShiLtJGpt-Prkdc^em26Cd52^Il2rg^em26Cd22^kit^em1Cin(V831M)^/GptCrl coisogenic immunodeficient mice (NCG-X), which supported human hematopoietic reconstitution without the need for irradiation (Fig. 4a) (Gu et al. 2022). 16 weeks post-transplantation, human engraftment was assessed in peripheral blood and isolated bone marrow. No significant differences in human chimerism were observed between the unedited and edited HSPCs in peripheral blood (21.18% ± 4.16% v.s. 14.95% ± 6.80%) and isolated bone marrow cells (90.70% ± 6.08% v.s. 85.49% ± 5.13%) of mice (Fig. 4b and Fig. S4). The self-renewal and erythroid differentiation potential of HSPCs were further analyzed via flow cytometry, showing comparable percentages of human HSPCs (CD45 + /CD34 + , 10.58% ± 2.30% v.s. 14.86% ± 4.00%) and erythroid cells (hCD235a + , 7.87% ± 2.30% v.s. 7.28% ± 3.17%) in the engrafted bone marrow of the unedited and edited group (Fig. 4c,d and Fig. S4). Notably, the HBB + cells were detected in 19.04% ± 0.67% of cells in the edited group, while none were detected in the unedited group (Fig. 4e, Fig. S4). The β-globin to α-globin ratio in the bone marrow 16 weeks post-transplantation significantly improved in the edited group compared to the unedited group, as confirmed by western blot analysis (Fig. S5). Additionally, the repair frequency in engrafted bone marrow cells was confirmed via NGS, indicating that editing efficiency was maintained from 21.4% at 96 h after editing to 17.21% ± 3.66% at 16 weeks after transplantation (Fig. 4 f,g).

The therapeutic gene correction in long-term HSCs was also evaluated at 26 weeks after xenotransplantation of other donor HSPCs. Both the unedited and edited donor HSPCs exhibited similar levels of human cell chimerism (68.34% ± 15.10% v.s. 64.81% ± 17.24%) in engrafted bone marrow cells at 26 weeks post-transplantation (Fig. S6a). The self-renewal capacity and multilineage hematopoietic repopulation potential of the edited HSPCs were assessed by multiparameter flow cytometric analysis of the engrafted bone marrow at 26 weeks after transplantation. The results demonstrated the successful reconstitution of HSPCs, T cells, B cells, myeloid cells, NK cells, and erythroid cells at comparable chimerism levels between the unedited and edited donor progeny cells (Fig. S6b). The proportion of HBB + cells in erythroid cells derived from edited donor HSPCs was maintained at 22.78% ± 19.81% at 26 weeks post-transplantation, declining from 52.85% ± 36.37% at 16 weeks post-transplantation (Fig. S6c). Compared to the unedited group, the β-globin to α-globin ratio in the bone marrow remained more balanced in most of the edited group 26 weeks post-transplantation (Fig. S6d,e). Similarly, the repair frequency in engrafted bone marrow cells decreased from 46% at 96 h after editing to 6.45% ± 6.32% at 26 weeks after xenotransplantation, with a maximal correction efficiency of 21.17% (Fig. S6f). This reduction was consistent with previous reports (DeWitt et al. 2016; Hoban et al. 2015; Wu et al. 2019) and may be attributed to the inherent resistance of stem cells to HDR-based editing or the potential impairment of long-term renewal capacity in HSCs caused by HDR.

In general, the gene correction of patient-derived HSPCs with homozygote *HBB* CD41-42 (-TCTT) mutation enhanced *HBB* expression in the progeny cells after xenotransplantation, with no evidence showing an impact on HSPCs renewal and multilineage hematopoietic repopulation, reinforcing its potential as a gene therapy for β-thalassemia.

### The genome-wide off-target analysis supported the safety of the gene correction strategy

**Fig. 5:**
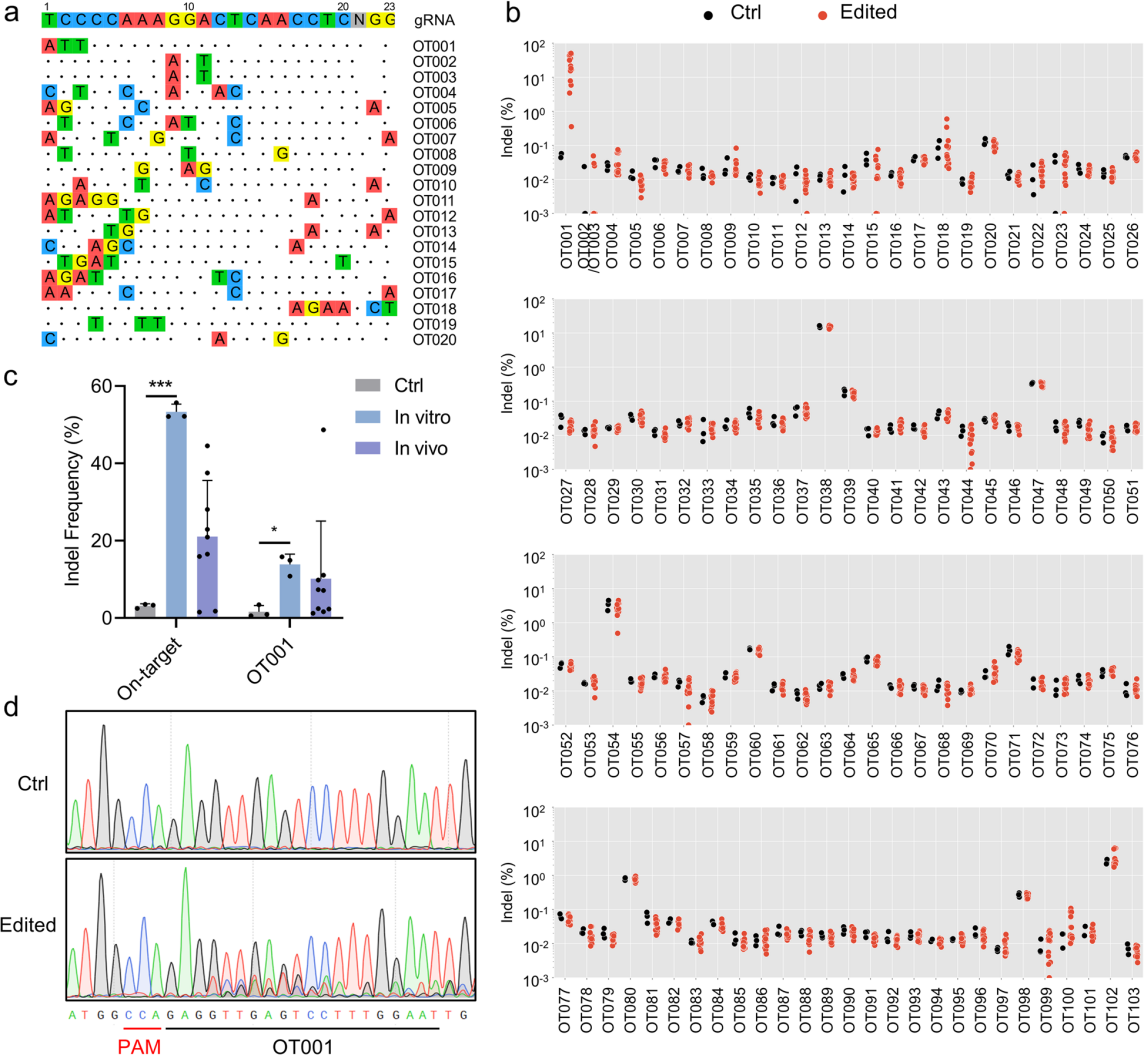
Off-target analysis of *HBB* CD41-42 (-TCTT) patient-derived CD34^+^ cells corrected by CRISPR/Cas9. **a** Overview of the top 20 candidate off-target sites for Cas9:sgRNA_1 RNP predicted by SITE-seq analysis and CRISPOR. The sequence of on-target sites and PAM was shown at the top. Dots represented matches to the on-target site, while the colored nucleotides represented mismatches. **b** The indel frequency at the 103 candidate off-target sites in both patient-derived HSPCs edited in vitro and the engrafted cells gathered from mice bone marrow 8 weeks or 16 weeks after xenotransplantation, which was detected by amplicon deep sequencing. A highly complementary site (chr3_23029106_T1P69, OT001) with definite off-target editing efficiency was shown. **c** The specific indel frequency of the on-target site and OT001 detected by amplicon deep sequencing. The Ctrl indicated the untransfected HSPCs. The In vitro indicated the patient-derived HSPCs edited in vitro. The In vivo indicated the engrafted cells gathered from mice bone marrow 8 weeks or 16 weeks after xenotransplantation. The data represent the mean ± SD for 3 to 9 replications. Each dot represented independent donor HSPCs or a single recipient mouse. ***, *P* < 0.001, *, *P* < 0.05 (by unpaired t-test). **d** Representative Sanger sequencing chromatographs showing the definite off-target editing efficiency at OT001 in the edited homozygote *HBB* CD41-42 (-TCTT) patient-derived CD34^+^ cells. Ctrl indicated the untransfected HSPCs, while Edited indicated the HSPCs transfected with Cas9:sgRNA_1 RNP and ssODNs as templates

To comprehensively evaluate the specificity of this CRISPR/Cas9-based gene therapy strategy, we evaluated the genome-wide activity of Cas9:sgRNA_1 RNP using the SITE-Seq, a method that can sensitively identify Cas9 cleavage sites in the purified genome and predict potential off-target sites (Cameron et al. 2017). The purified genome was treated with either 90 nmol or 256 nmol Cas9:sgRNA_1 RNP, and the resulting breakpoints were located through NGS (Table S2 and S3). Besides, we predicted the top off-target sites with fewer than 4 mismatches in silico via CRISPOR (Concordet & Haeussler 2018) (Table S4). Based on the results of SITE-Seq and bioinformatic prediction, we ultimately identified 103 candidate off-target sites (Fig. 5a and Fig. S7). Amplicon-sequencing was performed on both patient-derived HSPCs edited in vitro and the engrafted cells gathered from mice bone marrow 16 weeks after xenotransplantation, and a highly complementary site (chr3_23029106_T1P69, OT001) showed a significant off-target editing efficiency (12.89% ± 2.89% in vitro and 13.73% ± 19.87%) compared to the control group (1.64% ± 1.57%) (Fig. 5b,c). This off-target editing was further confirmed by Sanger sequencing (Fig. 5d). The identified off-target site is located in an intergenic region without overlapping with known regulatory or conserved sequence regions. These findings suggested that the sgRNA-dependent DNA off-target activity poses a minimal risk in the CRISPR/Cas9-mediated gene therapy targeting the *HBB* CD41-42 (-TCTT) mutation site.

## Discussion

Our study demonstrated effective correction of the *HBB* CD41-42 (-TCTT) mutation in patients-derived HSPCs can be achieved using the CRISPR/Cas9 system with ssODNs as donors. This approach resulted in improved *HBB* expression both in vitro and in vivo, without impairing the self-renewal and multilineage hematopoietic repopulation capacities of HSPCs. Recently, Yuxuan Wu’s team has reported a comparable gene editing approach using the same sgRNA, though without ssODNs, in patient-derived HSPCs from one donor with a homozygote CD41-42 (-TCTT) mutation and four donors with heterozygote mutations. The repair rates in the homozygous cells were approximately 22%−27% in vitro and 12%–15% in repopulating HSCs 4 months after transplantation (F. Yang et al. 2023). Our study focused on the correction of patient-derived HSPCs with homozygote CD41-42 (-TCTT) mutation, with three different donors’ HSPCs evaluated in vitro and two different donors’ HSPCs evaluated via xenotransplantation. Our study achieved more cells edited and higher editing efficiency in vitro and post-transplantation with similar amounts of Cas9. Furthermore, extended engraftment over 26 weeks confirmed the multi-lineage hematopoietic repopulation of the edited HSPCs. Notably, our study also identified a previously unreported gRNA-dependent off-target site, highlighting the need for further optimization of this CRISPR/Cas9-based gene therapy approach. Overall, the findings from both Yuxuan Wu's team and our study are complementary, advancing the gene therapy for the *HBB* CD41-42 (-TCTT) mutation. Since this mutation is the most common cause of β-thalassemia in China and Southeast Asia, this CRISPR/Cas9-based gene editing therapy in patients-derived HSPCs could potentially benefit tens of thousands of β-thalassemia patients (Huang et al. 1990; Lai et al. 2017).

Inefficient HDR has long been a significant challenge impeding CRISPR/Cas9-based precision gene editing therapy in HSPCs. In this study, the functional reparation frequency of homozygous *HBB* CD41-42 (-TCTT) in patient-derived HSPCs was 49.92% ± 4.31% in vitro without selection, surpassing the HDR efficiency reported in most existing studies (Azhagiri et al. 2021; Park et al. 2019; Pavel-Dinu et al. 2019; Vakulskas et al. 2018). The reparation efficiency was maintained at up to 21.17% 26 weeks after transplantation, with the proportion of HBB + cells in erythroid cells maintaining at 22.78% ± 19.81%, up to 70.30%. Previous studies have shown that patients undergoing allogeneic HSCT could achieve good condition and become transfusion-independent with a minimum of 20% donor-derived nucleated cells and 35% donor-derived burst-forming unit-erythroid colonies in the bone marrow (Andreani et al. 2011; King et al. 2015). Due to the improved survival of mature erythroid cells and rectification of inefficient erythropoiesis, the corrected cells exhibited a selective advantage in vivo (Miccio et al. 2008), suggesting that this CRISPR/Cas9-based gene editing therapy may provide clinical benefits with effective long-term engraftment.

Increasing the frequency of HDR-based edited cells in vivo following HSCT is crucial for optimizing the therapeutic effect, which depends on the engraftment of corrected long-term hematopoietic stem cells (LT-HSCs). However, it appears that the HDR editing efficiency and HSCs stemness are inherently imbalanced, possibly resulting from the quiescence and limited template uptake of LT-HSCs (S. Ferrari et al. 2022a, b; Shin et al. 2020). As a result, HDR-based editing may predominantly occur in hematopoietic progenitor cells or short-term HSCs, which are progressively lost after transplantation, leading to reduced gene editing efficiency in engrafted mouse bone marrow cells and inefficient long-term transplantation, as shown in this study (Azhagiri et al. 2021; Shin et al. 2020). Transient expression of adenovirus 5 E4orf6/7 protein to modify the HSPCs cell cycle and activate the HDR pathway, along with transient p53 inhibition to improve the viability of edited HSPCs, has been shown to improve HDR-based editing efficiency in long-term engraftment by up to 50% (Ferrari et al. 2020). Additionally, altering the HDR/NHEJ editing preferences in HSCs through the use of Cas9/HDR template conjugates or HDR-promoting factors might enhance the editing effectiveness of input HSPCs, which potentially benefit the edited cells in competition for the bone marrow niche(Allen, Kalter, Rosenberg, & Hendel, 2023; Azhagiri et al. 2021).

The safety of CRISPR/Cas9-mediated gene therapy remains a significant concern. Although the adeno-associated virus (AAV) was widely used as DNA repair templates in HSPCs, the rAAV sequences could integrate randomly into the host genome at low frequency, potentially involving in oncogenesis (Charlesworth et al. 2018; Dalwadi et al. 2021; Dever et al. 2016; Nguyen et al. 2020). AAV inverted terminal repeats (ITR) activated the p53-mediated DNA damage response, leading to cell-cycle arrest in HSPCs with AAV genome accrual and increased integration (Samuele Ferrari et al. 2022a, b). The combination of CRISPR/Cas9 and AAV6 in HSPCs has resulted in frequent concatemeric insertions at the target sites, causing imprecise repair and unexpected clinical outcomes (Suchy et al. 2024). As such, we opted to use ssODNs as DNA donors in this study to reduce the risk of genotoxic integration events. Besides, we systematically predicted the candidate off-target sites using SITE-seq and in silico analysis, one of which showed significant off-target editing. Although the off-target site is located in an intergenic region that is unlikely to cause gene disruption, further follow-up studies are necessary. In addition, systematic evaluation of other potential genotoxicities resulting from the CRISPR/Cas9-induced double-strand breaks, such as chromosomal disorganization, unpredictable large deletion, and genomic rearrangements, is essential (Kosicki et al. 2018; Leibowitz et al. 2021).A robust protocol for the quality control of edited HSPCs before transplantation is still needed, potentially involving whole-genome sequencing and global gene expression analysis(Leibowitz et al. 2021; Nahmad et al. 2022).

Taken together, this study proposed a CRISPR/Cas9-based gene therapy strategy to precisely correct *HBB* CD41-42 (-TCTT) mutations in patient-derived HSPCs with preclinical experimental support. For successful clinical translation, further studies are required to enhance the editing efficiency in LT-HSCs and to ensure both the efficacy and safety of the therapy.

## Supplementary Information

Supplementary Material 1.

Supplementary Material 2.

## Data Availability

Original data in the paper is available via contacting the corresponding author.

## References

[CR1] Adachi K, Konitzer P, Pang J, Reddy KS, Surrey S. Amino Acids Responsible for Decreased 2, 3-Biphosphosphoglycerate Binding to Fetal Hemoglobin. Blood. 1997;90(8):2916–20. 10.1182/blood.V90.8.2916. 9376571

[CR2] Allen, D., Kalter, N., Rosenberg, M., & Hendel, A. (2023). Homology-Directed-Repair-Based Genome Editing in HSPCs for the Treatment of Inborn Errors of Immunity and Blood Disorders. Pharmaceutics, 15(5). 10.3390/pharmaceutics15051329 10.3390/pharmaceutics15051329 PMC10220672 37242571

[CR3] Andreani, M., Testi, M., Gaziev, J., Condello, R., Bontadini, A., Tazzari, P. L., . . . Lucarelli, G. (2011). Quantitatively different red cell/nucleated cell chimerism in patients with long-term, persistent hematopoietic mixed chimerism after bone marrow transplantation for thalassemia major or sickle cell disease. Haematologica, 96(1), 128–133. 10.3324/haematol.2010.031013 10.3324/haematol.2010.031013 PMC3012776 20935000

[CR4] Azhagiri MKK, Babu P, Venkatesan V, Thangavel S. Homology-directed gene-editing approaches for hematopoietic stem and progenitor cell gene therapy. Stem Cell Res Ther. 2021;12(1):500. 10.1186/s13287-021-02565-6. 34503562 10.1186/s13287-021-02565-6 PMC8428126

[CR5] Cameron, P., Fuller, C. K., Donohoue, P. D., Jones, B. N., Thompson, M. S., Carter, M. M., . . . May, A. P. (2017). Mapping the genomic landscape of CRISPR-Cas9 cleavage. Nat Methods, 14(6), 600–606. 10.1038/nmeth.4284 10.1038/nmeth.4284 28459459

[CR6] Canver MC, Orkin SH. Customizing the genome as therapy for the beta-hemoglobinopathies. Blood. 2016;127(21):2536–45. 10.1182/blood-2016-01-678128. 27053533 10.1182/blood-2016-01-678128 PMC4882803

[CR7] Cao A, Kan YW. The prevention of thalassemia. Cold Spring Harb Perspect Med. 2013;3(2): a011775. 10.1101/cshperspect.a011775. 23378598 10.1101/cshperspect.a011775 PMC3552345

[CR8] Charlesworth, C. T., Camarena, J., Cromer, M. K., Vaidyanathan, S., Bak, R. O., Carte, J. M., . . . Porteus, M. H. (2018). Priming Human Repopulating Hematopoietic Stem and Progenitor Cells for Cas9/sgRNA Gene Targeting. Molecular Therapy - Nucleic Acids, 12, 89–104. 10.1016/j.omtn.2018.04.017 10.1016/j.omtn.2018.04.017 PMC6023838 30195800

[CR9] Clement, K., Rees, H., Canver, M. C., Gehrke, J. M., Farouni, R., Hsu, J. Y., . . . Pinello, L. (2019). CRISPResso2 provides accurate and rapid genome editing sequence analysis. Nat Biotechnol, 37(3), 224–226. 10.1038/s41587-019-0032-3 10.1038/s41587-019-0032-3 PMC6533916 30809026

[CR10] Concordet JP, Haeussler M. CRISPOR: intuitive guide selection for CRISPR/Cas9 genome editing experiments and screens. Nucleic Acids Res. 2018;46(W1):W242–5. 10.1093/nar/gky354. 29762716 10.1093/nar/gky354 PMC6030908

[CR11] Cong, L., Ran, F. A., Cox, D., Lin, S., Barretto, R., Habib, N., . . . Zhang, F. (2013). Multiplex genome engineering using CRISPR/Cas systems. Science, 339(6121), 819–823. 10.1126/science.1231143 10.1126/science.1231143 PMC3795411 23287718

[CR12] Cosenza LC, Gasparello J, Romanini N, Zurlo M, Zuccato C, Gambari R, Finotti A. Efficient CRISPR-Cas9-based genome editing of β-globin gene on erythroid cells from homozygous β039-thalassemia patients. Molecular Therapy - Methods & Clinical Development. 2021;21:507–23. 10.1016/j.omtm.2021.03.025. 33997100 10.1016/j.omtm.2021.03.025 PMC8091488

[CR13] Dalwadi DA, Calabria A, Tiyaboonchai A, Posey J, Naugler WE, Montini E, Grompe M. AAV integration in human hepatocytes. Mol Ther. 2021;29(10):2898–909. 10.1016/j.ymthe.2021.08.031. 34461297 10.1016/j.ymthe.2021.08.031 PMC8531150

[CR14] De Dreuzy, E., Heath, J., Zuris, J. A., Sousa, P., Viswanathan, R., Scott, S., . . . Chang, K.-H. (2019). EDIT-301: An Experimental Autologous Cell Therapy Comprising Cas12a-RNP Modified mPB-CD34+ Cells for the Potential Treatment of SCD. Blood, 134(Supplement_1), 4636–4636. 10.1182/blood-2019-130256

[CR15] Dever, D. P., Bak, R. O., Reinisch, A., Camarena, J., Washington, G., Nicolas, C. E., . . . Porteus, M. H. (2016). CRISPR/Cas9 beta-globin gene targeting in human haematopoietic stem cells. Nature, 539(7629), 384–389. 10.1038/nature20134 10.1038/nature20134 PMC5898607 27820943

[CR16] DeWitt, M. A., Magis, W., Bray, N. L., Wang, T., Berman, J. R., Urbinati, F., . . . Corn, J. E. (2016). Selection-free genome editing of the sickle mutation in human adult hematopoietic stem/progenitor cells. Sci Transl Med, 8(360), 360ra134. 10.1126/scitranslmed.aaf9336 10.1126/scitranslmed.aaf9336 PMC5500303 27733558

[CR17] Ferrari, S., Jacob, A., Beretta, S., Unali, G., Albano, L., Vavassori, V., . . . Naldini, L. (2020). Efficient gene editing of human long-term hematopoietic stem cells validated by clonal tracking. Nat Biotechnol, 38(11), 1298–1308. 10.1038/s41587-020-0551-y 10.1038/s41587-020-0551-y PMC7610558 32601433

[CR18] Ferrari, S., Jacob, A., Cesana, D., Laugel, M., Beretta, S., Varesi, A., . . . Naldini, L. (2022). Choice of template delivery mitigates the genotoxic risk and adverse impact of editing in human hematopoietic stem cells. Cell Stem Cell, 29(10), 1428–1444 e1429. 10.1016/j.stem.2022.09.001 10.1016/j.stem.2022.09.001 PMC9550218 36206730

[CR19] Ferrari, S., Jacob, A., Cesana, D., Laugel, M., Beretta, S., Varesi, A., . . . Naldini, L. (2022). Choice of template delivery mitigates the genotoxic risk and adverse impact of editing in human hematopoietic stem cells. Cell Stem Cell, 29(10), 1428–1444.e1429. 10.1016/j.stem.2022.09.001 10.1016/j.stem.2022.09.001 PMC9550218 36206730

[CR20] Frangoul, H., Altshuler, D., Cappellini, M. D., Chen, Y. S., Domm, J., Eustace, B. K., . . . Corbacioglu, S. (2021). CRISPR-Cas9 Gene Editing for Sickle Cell Disease and beta-Thalassemia. N Engl J Med, 384(3), 252–260. 10.1056/NEJMoa2031054 10.1056/NEJMoa2031054 33283989

[CR21] Frangoul, H., Locatelli, F., Bhatia, M., Mapara, M. Y., Molinari, L., Sharma, A., . . . Grupp, S. (2022). Efficacy and Safety of a Single Dose of Exagamglogene Autotemcel for Severe Sickle Cell Disease. Blood, 140(Supplement 1), 29–31. 10.1182/blood-2022-162353

[CR22] Fu, B., Liao, J., Chen, S., Li, W., Wang, Q., Hu, J., . . . Wu, Y. (2022). CRISPR-Cas9-mediated gene editing of the BCL11A enhancer for pediatric beta(0)/beta(0) transfusion-dependent beta-thalassemia. Nat Med, 28(8), 1573–1580. 10.1038/s41591-022-01906-z 10.1038/s41591-022-01906-z 35922667

[CR23] Galanello R, Origa R. Beta-thalassemia. Orphanet J Rare Dis. 2010;5:11. 10.1186/1750-1172-5-11. 20492708 10.1186/1750-1172-5-11 PMC2893117

[CR24] Gillmore, J. D., Gane, E., Taubel, J., Kao, J., Fontana, M., Maitland, M. L., . . . Lebwohl, D. (2021). CRISPR-Cas9 In Vivo Gene Editing for Transthyretin Amyloidosis. N Engl J Med, 385(6), 493–502. 10.1056/NEJMoa2107454 10.1056/NEJMoa2107454 34215024

[CR25] Gu, T., Ju, C., Sun, H., Gao, X., Zhang, M., Yu, W., . . . Zhao, J. (2022). Abstract 5621: NCG-X mouse: A novel animal model to evaluate preclinical studies of humanized erythroid reconstitution without irradiation. Cancer Research, 82(12_Supplement), 5621–5621. 10.1158/1538-7445.Am2022-5621

[CR26] Han, L., He, H., Yang, Y., Meng, Q., Ye, F., Chen, G., & Zhang, J. (2021). Distinctive Clinical and Pathologic Features of Immature Teratomas Arising From Induced Pluripotent Stem Cell Injection in a Patient With Type 2 Diabetes. 10.21203/rs.3.rs-580493/v1 10.1089/scd.2021.0255 35018826

[CR27] Hoban, M. D., Cost, G. J., Mendel, M. C., Romero, Z., Kaufman, M. L., Joglekar, A. V., . . . Kohn, D. B. (2015). Correction of the sickle cell disease mutation in human hematopoietic stem/progenitor cells. Blood, 125(17), 2597–2604. 10.1182/blood-2014-12-615948 10.1182/blood-2014-12-615948 PMC4408287 25733580

[CR28] Huang SZ, Zhou XD, Zhu H, Ren ZR, Zeng YT. Detection of beta-thalassemia mutations in the Chinese using amplified DNA from dried blood specimens. Hum Genet. 1990;84(2):129–31. 10.1007/BF00208926. 2298448 10.1007/BF00208926

[CR29] Jinek M, Chylinski K, Fonfara I, Hauer M, Doudna JA, Charpentier E. A programmable dual-RNA-guided DNA endonuclease in adaptive bacterial immunity. Science. 2012;337(6096):816–21. 10.1126/science.1225829. 22745249 10.1126/science.1225829 PMC6286148

[CR30] Kass EM, Jasin M. Collaboration and competition between DNA double-strand break repair pathways. FEBS Lett. 2010;584(17):3703–8. 10.1016/j.febslet.2010.07.057. 20691183 10.1016/j.febslet.2010.07.057 PMC3954739

[CR31] Kimura A, Matsunaga E, Takihara Y, Nakamura T, Takagi Y, Lin S, Lee H. Structural analysis of a beta-thalassemia gene found in Taiwan. J Biol Chem. 1983;258(5):2748–9. 10.1016/s0021-9258(18)32776-5. 6826539

[CR32] King, A. A., Kamani, N., Bunin, N., Sahdev, I., Brochstein, J., Hayashi, R. J., . . . Shenoy, S. (2015). Successful matched sibling donor marrow transplantation following reduced intensity conditioning in children with hemoglobinopathies. Am J Hematol, 90(12), 1093–1098. 10.1002/ajh.24183 10.1002/ajh.24183 26348869

[CR33] Kosicki M, Tomberg K, Bradley A. Repair of double-strand breaks induced by CRISPR-Cas9 leads to large deletions and complex rearrangements. Nat Biotechnol. 2018;36(8):765–71. 10.1038/nbt.4192. 30010673 10.1038/nbt.4192 PMC6390938

[CR34] Kountouris P, Lederer CW, Fanis P, Feleki X, Old J, Kleanthous M. IthaGenes: an interactive database for haemoglobin variations and epidemiology. PLoS ONE. 2014;9(7): e103020. 10.1371/journal.pone.0103020. 25058394 10.1371/journal.pone.0103020 PMC4109966

[CR35] Kurita, R., Suda, N., Sudo, K., Miharada, K., Hiroyama, T., Miyoshi, H., . . . Nakamura, Y. (2013). Establishment of immortalized human erythroid progenitor cell lines able to produce enucleated red blood cells. PLoS One, 8(3), e59890. 10.1371/journal.pone.0059890 10.1371/journal.pone.0059890 PMC3606290 23533656

[CR36] Lai K, Huang G, Su L, He Y. The prevalence of thalassemia in mainland China: evidence from epidemiological surveys. Sci Rep. 2017;7(1):920. 10.1038/s41598-017-00967-2. 28424478 10.1038/s41598-017-00967-2 PMC5430438

[CR37] Langer, A. L. (1993). Beta-Thalassemia. In M. P. Adam, G. M. Mirzaa, R. A. Pagon, S. E. Wallace, L. J. H. Bean, K. W. Gripp, & A. Amemiya (Eds.), GeneReviews((R)). Seattle (WA). 20301599

[CR38] Laosombat, V., Wongchanchailert, M., Sattayasevana, B., Wiriyasateinkul, A., & Fucharoen, S. (2001). Clinical and hematologic features of beta0-thalassemia (frameshift 41/42 mutation) in Thai patients. Haematologica, 86(2), 138–141. Retrieved from https://www.ncbi.nlm.nih.gov/pubmed/11224481 11224481

[CR39] Leibowitz, M. L., Papathanasiou, S., Doerfler, P. A., Blaine, L. J., Sun, L., Yao, Y., . . . Pellman, D. (2021). Chromothripsis as an on-target consequence of CRISPR-Cas9 genome editing. Nat Genet, 53(6), 895–905. 10.1038/s41588-021-00838-7 10.1038/s41588-021-00838-7 PMC8192433 33846636

[CR40] Liang, P., Xu, Y., Zhang, X., Ding, C., Huang, R., Zhang, Z., . . . Huang, J. (2015). CRISPR/Cas9-mediated gene editing in human tripronuclear zygotes. Protein Cell, 6(5), 363–372. 10.1007/s13238-015-0153-5 10.1007/s13238-015-0153-5 PMC4417674 25894090

[CR41] Lieber MR. The mechanism of human nonhomologous DNA end joining. J Biol Chem. 2008;283(1):1–5. 10.1074/jbc.R700039200. 17999957 10.1074/jbc.R700039200

[CR42] Liu, Y., Yang, Y., Kang, X., Lin, B., Yu, Q., Song, B., . . . Fan, Y. (2017). One-Step Biallelic and Scarless Correction of a beta-Thalassemia Mutation in Patient-Specific iPSCs without Drug Selection. Mol Ther Nucleic Acids, 6, 57–67. 10.1016/j.omtn.2016.11.010 10.1016/j.omtn.2016.11.010 PMC5363452 28325300

[CR43] Liu, R., Xu, H., Liang, J., Xie, W., Yang, G., Shi, L., . . . Lai, Y. (2022). Preliminary Result of the Safety and Efficacy of Autologous HBG1/2 Promoter-Modified CD34+ Hematopoietic Stem and Progenitor Cells (RM-001) in Transfusion-Dependent Βeta-Thalassemia. Blood, 140(Supplement 1), 4915–4916. 10.1182/blood-2022-169151

[CR44] Locatelli, F., Lang, P., Li, A., Corbacioglu, S., de la Fuente, J., Wall, D. A., . . . Frangoul, H. (2022). Efficacy and Safety of a Single Dose of Exagamglogene Autotemcel for Transfusion-Dependent β-Thalassemia. Blood, 140(Supplement 1), 4899–4901. 10.1182/blood-2022-166881

[CR45] Locatelli, F., Thompson, A. A., Kwiatkowski, J. L., Porter, J. B., Thrasher, A. J., Hongeng, S., . . . Walters, M. C. (2022). Betibeglogene Autotemcel Gene Therapy for Non-beta(0)/beta(0) Genotype beta-Thalassemia. N Engl J Med, 386(5), 415–427. 10.1056/NEJMoa2113206 10.1056/NEJMoa2113206 34891223

[CR46] Magrin, E., Semeraro, M., Hebert, N., Joseph, L., Magnani, A., Chalumeau, A., . . . Cavazzana, M. (2022). Long-term outcomes of lentiviral gene therapy for the beta-hemoglobinopathies: the HGB-205 trial. Nat Med, 28(1), 81–88. 10.1038/s41591-021-01650-w 10.1038/s41591-021-01650-w 35075288

[CR47] Miccio, A., Cesari, R., Lotti, F., Rossi, C., Sanvito, F., Ponzoni, M., . . . Ferrari, G. (2008). In vivo selection of genetically modified erythroblastic progenitors leads to long-term correction of beta-thalassemia. Proc Natl Acad Sci U S A, 105(30), 10547–10552. 10.1073/pnas.0711666105 10.1073/pnas.0711666105 PMC2492493 18650378

[CR48] Moynahan ME, Jasin M. Mitotic homologous recombination maintains genomic stability and suppresses tumorigenesis. Nat Rev Mol Cell Biol. 2010;11(3):196–207. 10.1038/nrm2851. 20177395 10.1038/nrm2851 PMC3261768

[CR49] Nahmad, A. D., Reuveni, E., Goldschmidt, E., Tenne, T., Liberman, M., Horovitz-Fried, M., . . . Barzel, A. (2022). Frequent aneuploidy in primary human T cells after CRISPR-Cas9 cleavage. Nat Biotechnol, 40(12), 1807–1813. 10.1038/s41587-022-01377-0 10.1038/s41587-022-01377-0 PMC7613940 35773341

[CR50] Nguyen, G. N., Everett, J. K., Kafle, S., Roche, A. M., Raymond, H. E., Leiby, J., . . . Sabatino, D. E. (2020). A long-term study of AAV gene therapy in dogs with hemophilia A identifies clonal expansions of transduced liver cells. Nature Biotechnology, 39(1), 47–55. 10.1038/s41587-020-0741-7 10.1038/s41587-020-0741-7 PMC7855056 33199875

[CR51] Niu, X., He, W., Song, B., Ou, Z., Fan, D., Chen, Y., . . . Sun, X. (2016). Combining Single Strand Oligodeoxynucleotides and CRISPR/Cas9 to Correct Gene Mutations in beta-Thalassemia-induced Pluripotent Stem Cells. J Biol Chem, 291(32), 16576–16585. 10.1074/jbc.M116.719237 10.1074/jbc.M116.719237 PMC4974373 27288406

[CR52] Papapetrou EP. Gene and Cell Therapy for beta-Thalassemia and Sickle Cell Disease with Induced Pluripotent Stem Cells (iPSCs): The Next Frontier. Adv Exp Med Biol. 2017;1013:219–40. 10.1007/978-1-4939-7299-9_9. 29127683 10.1007/978-1-4939-7299-9_9

[CR53] Park, S. H., Lee, C. M., Dever, D. P., Davis, T. H., Camarena, J., Srifa, W., . . . Bao, G. (2019). Highly efficient editing of the beta-globin gene in patient-derived hematopoietic stem and progenitor cells to treat sickle cell disease. Nucleic Acids Res, 47(15), 7955–7972. 10.1093/nar/gkz475 10.1093/nar/gkz475 PMC6735704 31147717

[CR54] Pavel-Dinu, M., Wiebking, V., Dejene, B. T., Srifa, W., Mantri, S., Nicolas, C. E., . . . Porteus, M. H. (2019). Gene correction for SCID-X1 in long-term hematopoietic stem cells. Nat Commun, 10(1), 1634. 10.1038/s41467-019-09614-y 10.1038/s41467-019-09614-y PMC6456568 30967552

[CR55] Shin, J. J., Schroder, M. S., Caiado, F., Wyman, S. K., Bray, N. L., Bordi, M., . . . Corn, J. E. (2020). Controlled Cycling and Quiescence Enables Efficient HDR in Engraftment-Enriched Adult Hematopoietic Stem and Progenitor Cells. Cell Rep, 32(9), 108093. 10.1016/j.celrep.2020.108093 10.1016/j.celrep.2020.108093 PMC7487781 32877675

[CR56] Smith, A. R., Schiller, G. J., Vercellotti, G. M., Kwiatkowski, J. L., Krishnamurti, L., Esrick, E. B., . . . Walters, M. C. (2019). Preliminary Results of a Phase 1/2 Clinical Study of Zinc Finger Nuclease-Mediated Editing of BCL11A in Autologous Hematopoietic Stem Cells for Transfusion-Dependent Beta Thalassemia. Blood, 134(Supplement_1), 3544–3544. 10.1182/blood-2019-125743

[CR57] Suchy, F. P., Karigane, D., Nakauchi, Y., Higuchi, M., Zhang, J., Pekrun, K., . . . Nakauchi, H. (2024). Genome engineering with Cas9 and AAV repair templates generates frequent concatemeric insertions of viral vectors. Nature Biotechnology. 10.1038/s41587-024-02171-w 10.1038/s41587-024-02171-w PMC11524221 38589662

[CR58] Taher AT, Weatherall DJ, Cappellini MD. Thalassaemia Lancet. 2018;391(10116):155–67. 10.1016/S0140-6736(17)31822-6. 28774421 10.1016/S0140-6736(17)31822-6

[CR59] Taher AT, Musallam KM, Cappellini MD. beta-Thalassemias. N Engl J Med. 2021;384(8):727–43. 10.1056/NEJMra2021838. 33626255 10.1056/NEJMra2021838

[CR60] Thompson, A. A., Walters, M. C., Kwiatkowski, J., Rasko, J. E. J., Ribeil, J. A., Hongeng, S., . . . Cavazzana, M. (2018). Gene Therapy in Patients with Transfusion-Dependent beta-Thalassemia. N Engl J Med, 378(16), 1479–1493. 10.1056/NEJMoa1705342 10.1056/NEJMoa1705342 29669226

[CR61] Vakulskas, C. A., Dever, D. P., Rettig, G. R., Turk, R., Jacobi, A. M., Collingwood, M. A., . . . Behlke, M. A. (2018). A high-fidelity Cas9 mutant delivered as a ribonucleoprotein complex enables efficient gene editing in human hematopoietic stem and progenitor cells. Nat Med, 24(8), 1216–1224. 10.1038/s41591-018-0137-0 10.1038/s41591-018-0137-0 PMC6107069 30082871

[CR62] Wei, D., Li, Y., Li, C., Peng, Z., Zhao, Y., Zhang, W., . . . Fang, R. (2019). Manufacturing Scale-up and Preclinical Development of ET-01, Autologous CD34+ Cells with the BCL11A Erythroid Enhancer Edited By CRISPR/Cas9, for Patients with β-Thalassemia Major. Blood, 134(Supplement_1), 965–965. 10.1182/blood-2019-126499

[CR63] Wen, J., Cao, T., Wu, J., Chen, Y., Zhi, S., Huang, Y., . . . Huang, J. (2022). Single AAV-mediated CRISPR-Nme2Cas9 efficiently reduces mutant hTTR expression in a transgenic mouse model of transthyretin amyloidosis. Mol Ther, 30(1), 164–174. 10.1016/j.ymthe.2021.05.010 10.1016/j.ymthe.2021.05.010 PMC8753293 33992807

[CR64] Wienert B, Martyn GE, Funnell APW, Quinlan KGR, Crossley M. Wake-up Sleepy Gene: Reactivating Fetal Globin for beta-Hemoglobinopathies. Trends Genet. 2018;34(12):927–40. 10.1016/j.tig.2018.09.004. 30287096 10.1016/j.tig.2018.09.004

[CR65] Wu, Y., Zeng, J., Roscoe, B. P., Liu, P., Yao, Q., Lazzarotto, C. R., . . . Bauer, D. E. (2019). Highly efficient therapeutic gene editing of human hematopoietic stem cells. Nat Med, 25(5), 776–783. 10.1038/s41591-019-0401-y 10.1038/s41591-019-0401-y PMC6512986 30911135

[CR66] Xian, Y., Xie, Y., Song, B., Ou, Z., Ouyang, S., Xie, Y., . . . Sun, X. (2020). The safety and effectiveness of genetically corrected iPSCs derived from beta-thalassaemia patients in nonmyeloablative beta-thalassaemic mice. Stem Cell Res Ther, 11(1), 288. 10.1186/s13287-020-01765-w 10.1186/s13287-020-01765-w PMC7367314 32678022

[CR67] Yang, Y., Zhang, X., Yi, L., Hou, Z., Chen, J., Kou, X., . . . Gao, S. (2016). Naive Induced Pluripotent Stem Cells Generated From beta-Thalassemia Fibroblasts Allow Efficient Gene Correction With CRISPR/Cas9. Stem Cells Transl Med, 5(1), 8–19. 10.5966/sctm.2015-0157 10.5966/sctm.2015-0157 PMC4704878 26676643

[CR68] Yang, F., Wang, Y., Wang, Q., Pang, J., Liu, G., Yang, Y., . . . Wu, Y. (2023). Efficient repair of human genetic defect by CRISPR/Cas9-mediated interlocus gene conversion. Life Medicine, 2(5). 10.1093/lifemedi/lnad042 10.1093/lifemedi/lnad042 PMC11749481 39872888

[CR69] Zhang, W., Cai, W. W., Zhou, W. P., Li, H. P., Li, L., Yan, W., . . . Xu, X. M. (2008). Evidence of gene conversion in the evolutionary process of the codon 41/42 (-CTTT) mutation causing beta-thalassemia in southern China. J Mol Evol, 66(5), 436–445. 10.1007/s00239-008-9096-2 10.1007/s00239-008-9096-2 18414926

[CR70] Zittersteijn HA, Harteveld CL, Klaver-Flores S, Lankester AC, Hoeben RC, Staal FJT, Goncalves M. A Small Key for a Heavy Door: Genetic Therapies for the Treatment of Hemoglobinopathies. Front Genome Ed. 2020;2: 617780. 10.3389/fgeed.2020.617780. 34713239 10.3389/fgeed.2020.617780 PMC8525365

