# Supplementary material for: Therapeutic gene correction of HBB frameshift CD41-42 (-TCTT) deletion in human hematopoietic stem cells
Source: Adv Biotechnol (Singap). 2025 Jan 2;3(1):2. doi: 10.1007/s44307-024-00053-5 (PMC11740860; doi:10.1007/s44307-024-00053-5)
Supplement: Supplementary file 2 — Supplementary Material 2. [file 44307_2024_53_MOESM2_ESM.pdf]

Therapeutic gene correction of *HBB* frameshift CD41-42 (-TCTT) deletion in human hematopoietic stem cells

Qianyi Liu,[1,#] Xinyu Li,[2,#] Hui Xu,[3,#] Ying Luo,[3] Lin Cheng,[3] Junbin Liang,[3] Yuelin He,[4] Haiying Liu,[1] Jianpei Fang,[2,*] Junjiu Huang[1,5,*]

[1]MOE Key Laboratory of Gene Function and Regulation, State Key Laboratory of Biocontrol, School of Life Sciences, Sun Yat-sen University, Guangzhou, Guangdong, 510275, China.

[2]Department of Pediatrics, Sun Yat-sen Memorial Hospital, Sun Yat-sen University, No.107, West Yan Jiang Road, Guangzhou, Guangdong, 510120, China.

[3]Reforgene Medicine, Guangzhou, Guangdong, 510535, China

[4]Dongguan Taixin Hospital, Dongguan, Guangdong, 523170, China

[5]Key Laboratory of Reproductive Medicine of Guangdong Province, the First Affiliated Hospital and School of Life Sciences, Sun Yat-sen University, Guangzhou, Guangdong, 510275, China.

[#]These authors contributed equally.

[*]Correspondence should be addressed to Huang Junjiu (hjunjiu@mail.sysu.edu.cn) and Jianpei Fang (fangjpei@mail.sysu.edu.cn)

We would like to submit this manuscript as an article in Advanced Biotechnology.

Supplemental Data

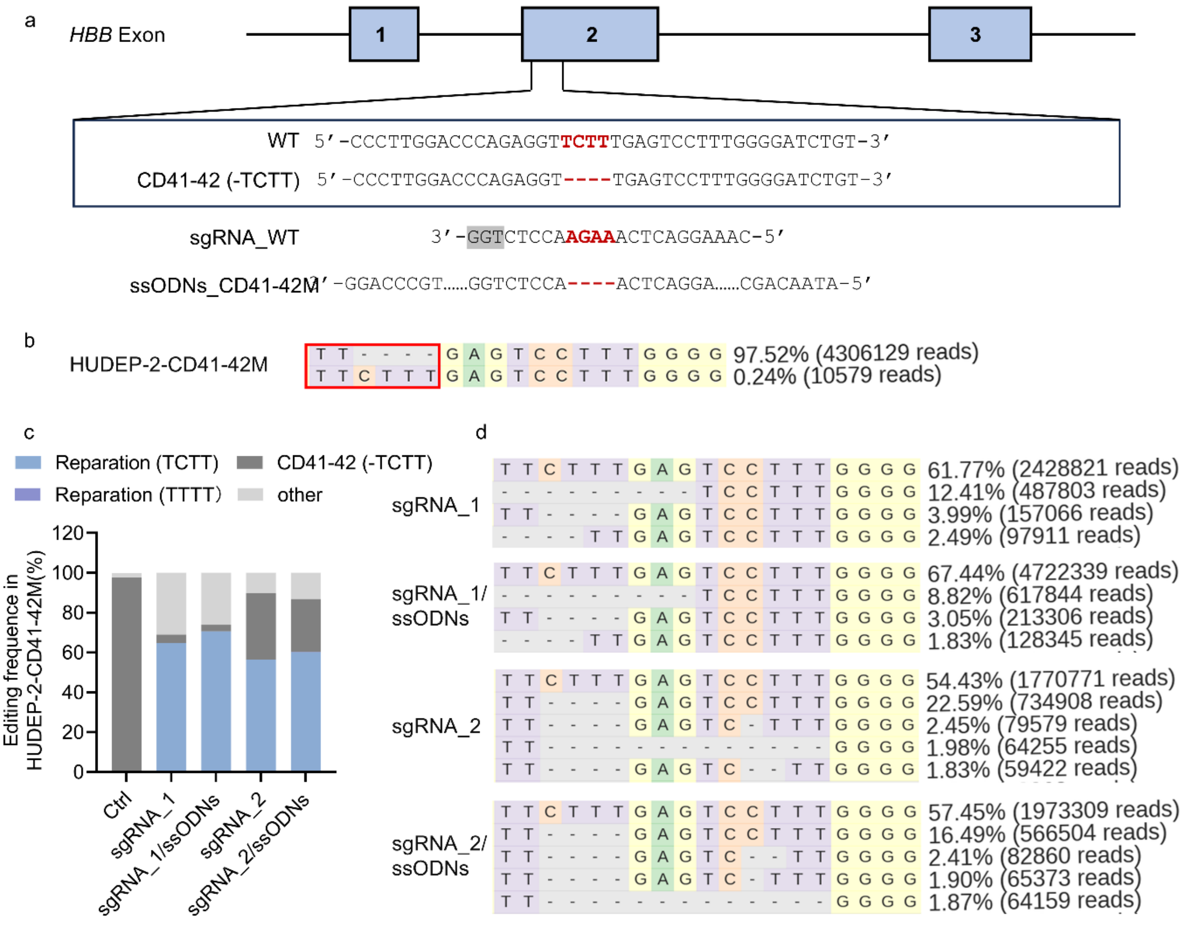

Fig. S1 Generation of *HBB* CD41-42 (-TCTT) mutant stable HUDEP-2 cell lines.

(a) Schematic of the generation of the HUDEP-2-CD41-42M cell line with the homozygotes *HBB* CD41-42 (-TCTT) mutation. The exons of *HBB* were labeled with blue boxes. The *HBB* CD41-42 (-TCTT) mutation was indicated in red. The sequences of sgRNA_WT for the generation of the HUDEP-2-CD41-42M cell line were shown in the middle with the PAM sequence shown in grey background. The ssODNs_CD41-42M for the generation of the HUDEP-2-CD41-42M cell line are shown below. (b) NGS data shows clear 4 bp deletions at the *HBB* CD41-42 sites, indicating the successful generation of the HUDEP-2-CD41-42M cell line. (c) NGS Data showing the reparation frequency of *HBB* CD41-42 (-TCTT) mutation in the HUDEP-2-CD41-42M cell line (including the samples in Fig. 1C), mediated by different sgRNAs (sgRNA_1 or sgRNA_2) with or without

ssODNs. Ctrl, HUDEP-2-CD41-42M cells that were not transfected. (d) Representative DNA profiles of HUDEP-2-CD41-42M cell line detected by NGS after transfected with Cas9:sgRNA_1 or Cas9:sgRNA_2, with or without ssODNs as templates.

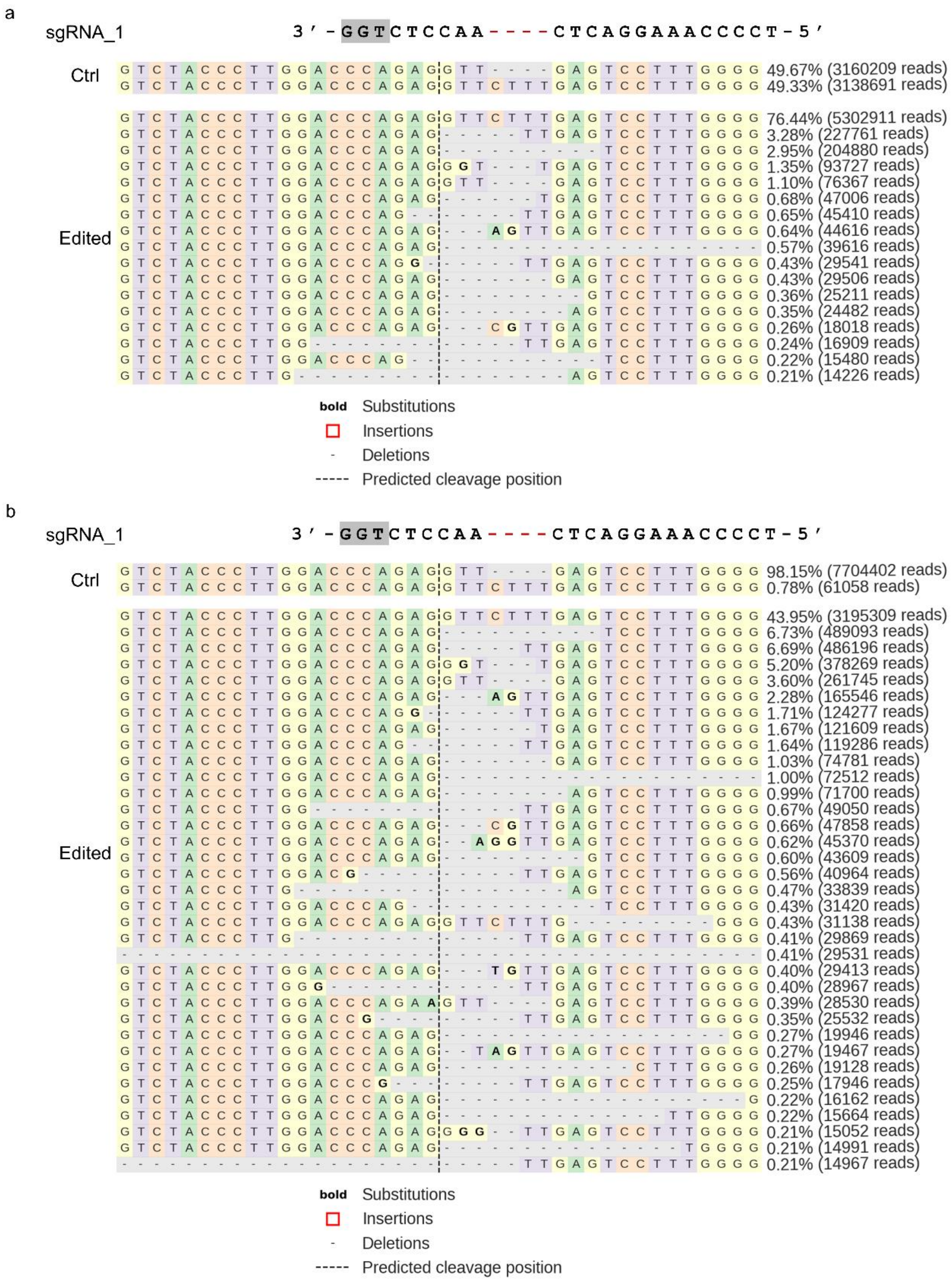

Fig. S2 Examples of NGS data showing correcting efficiency in heterozygote or homozygote *HBB* CD41-42 (-TCTT) patient-derived CD34^{+} cells.

(a) Representative DNA profiles of heterozygote *HBB* CD41-42 (-TCTT) patient-derived CD34+ cells detected by NGS 96 h after transfected with Cas9:sgRNA_1 and ssODNs as templates. (b)

Representative DNA profiles of homozygote *HBB* CD41-42 (-TCTT) patient-derived CD34+ cells detected by NGS 96 h after transfected with Cas9:sgRNA_1 and ssODNs as templates. The sequence of sgRNA_1 was shown at the top with the PAM sequence shown in grey background and the *HBB* CD41-42 (-TCTT) mutation shown in red. The bordered sequences indicated the substitutions. The red boxes indicated the insertions. The short line indicated the deletions. The vertical dotted line indicated the predicted cleavage position.

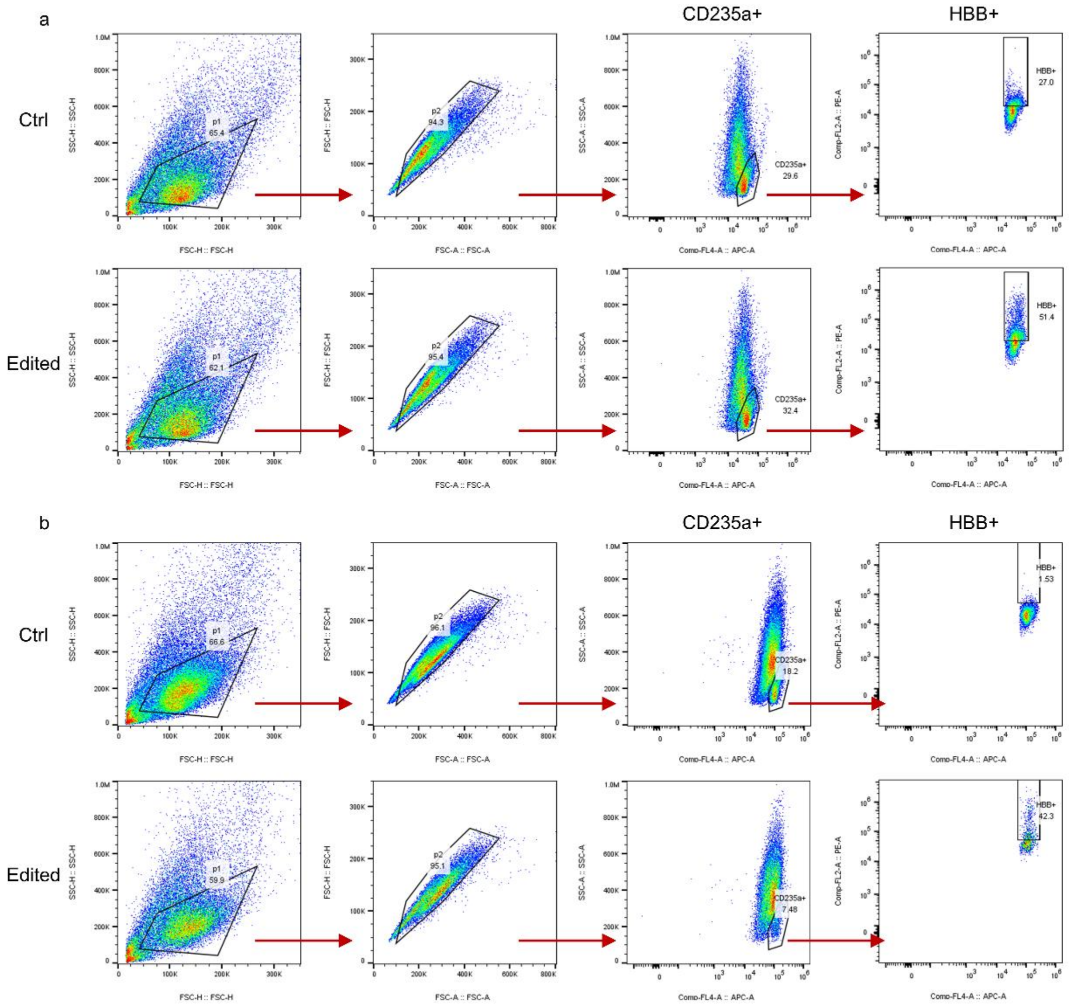

Fig. S3 Representative FACS plots of cultured erythroid cells after erythroid differentiation of patient-derived $CD34^+$ cells *in vitro*.

(a) FACS plots of the progeny cells after erythroid differentiation of heterozygote *HBB* CD41-42 (-TCTT) mutation patient-derived CD34+ cells (41-42/654) *in vitro* were shown as representative. (b) FACS plots of the progeny cell after erythroid differentiation of homozygote *HBB* CD41-42 (-TCTT) mutation patient-derived CD34+ cells (41-42/41-$42_{\#2}$) *in vitro* were shown as representative. Erythroid maturation was assessed by flow cytometry on the hCD235a+ fraction. The percentage of HBB+ cells in the hCD235a+ population was also analyzed. Ctrl indicated the untransfected HSPCs. Edited indicated the HSPCs transfected with Cas9:sgRNA_1 RNP and ssODNs as templates.

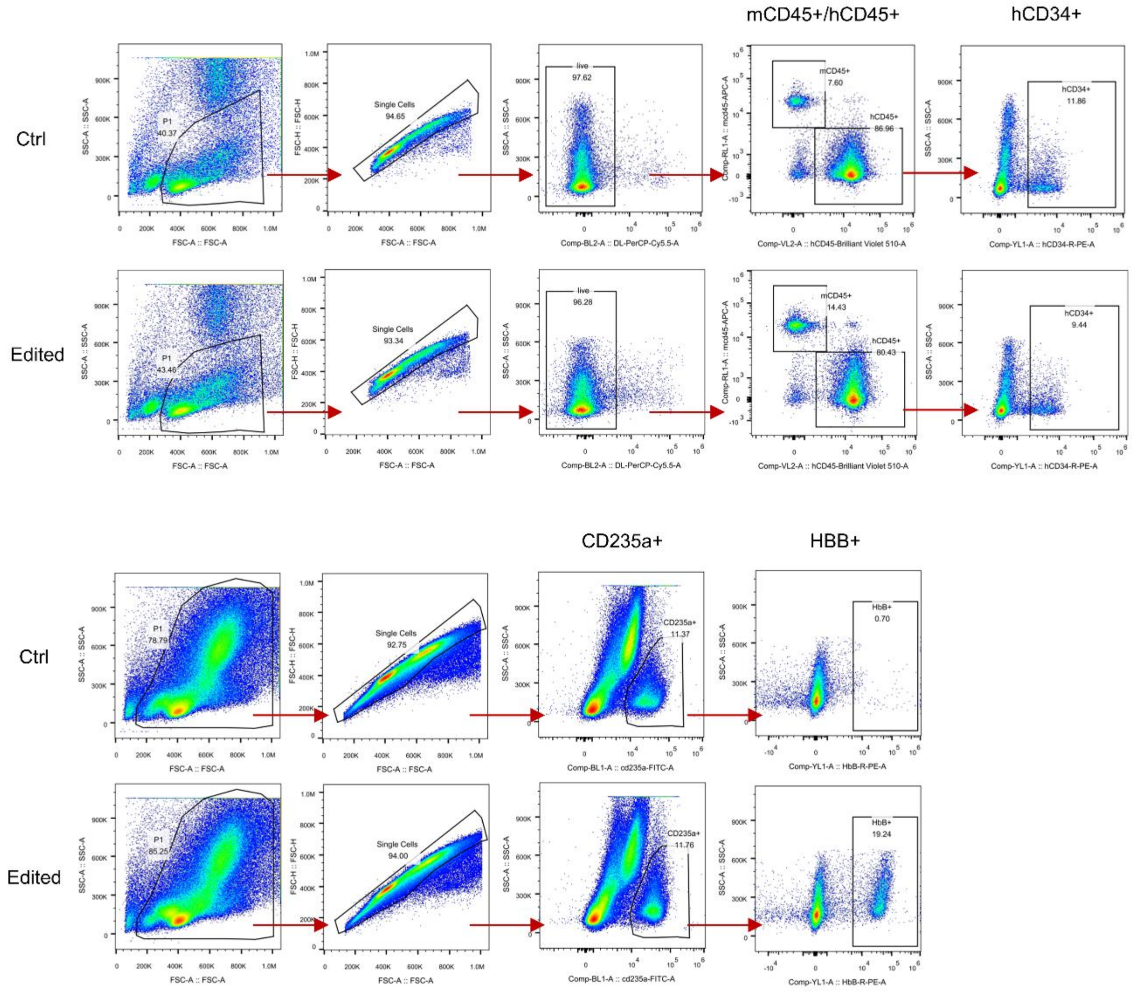

Fig. S4 Representative FACS plots of progeny cells from NCG-X mice after xenotransplantation of patient-derived CD34+ cells.

Representative FACS plots of the progeny cells in the engrafted mice bone marrow 16 weeks after xenotransplantation. The percentage of human cells (hCD45+), HSPCs (hCD45+/hCD34+), erythroid cells (hCD235a+), and the HBB+ cells (hCD235a+/HBB+) population was analyzed by flow cytometry.

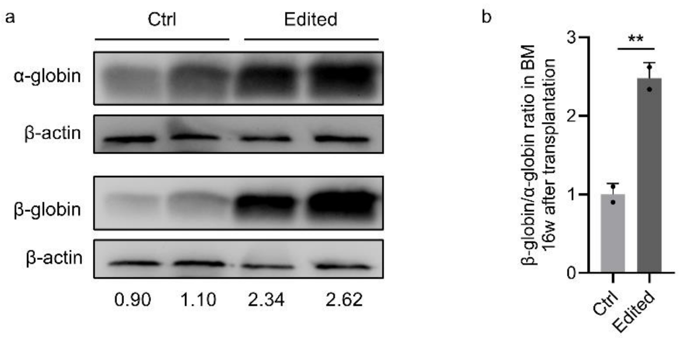

Fig. S5 Correcting *HBB* CD41-42 (-TCTT) mutation in patient-derived CD34+ cells improved the β-globin expression in the bone marrow of engrafted mice 16 weeks post-transplantation.

(a) Western blot analysis of the α-globin and β-globin expression in the bone marrow of engrafted mice 16 weeks after transplantation, with β-actin served as an internal control to relative expression analysis. The β-globin/α-globin ratio normalized to the average ratio in the control group is shown below. (b) Statistics of the β-globin/α-globin ratio in the bone marrow of engrafted mice 16 weeks after transplantation, as in (a), normalized to the average ratio in the control group. The full uncropped blot images were given in the Online Resource. Ctrl indicated the mice transplanted with untransfected HSPCs, while Edited indicated the mice transplanted with HSPCs transfected with Cas9:sgRNA_1 RNP and ssODNs as templates. **, $P < 0.01$ (by unpaired *t* test).

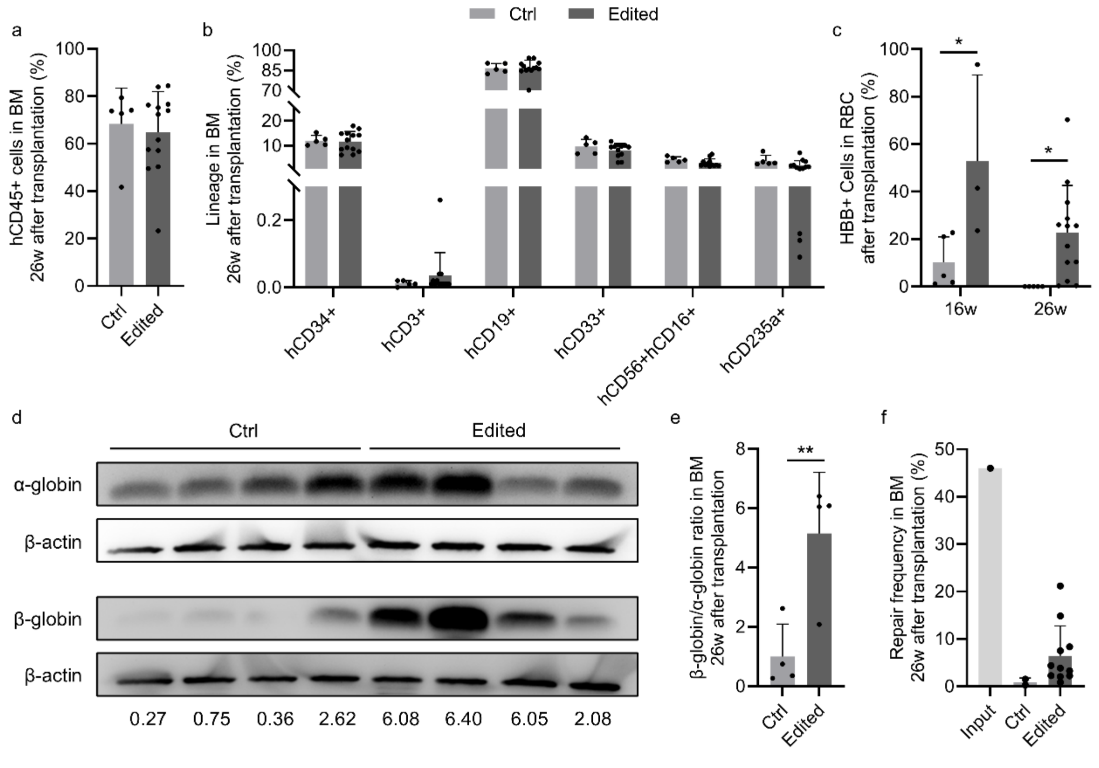

Fig. S6 Repopulation and effectiveness of patient-derived gene-edited CD34^{+} cells 26w after xenotransplantation.

(a) The human engraftment in the bone marrow of engrafted mice 26 weeks after transplantation, shown as the percentage of hCD45+ cells within the population of CD45+ cells (mouse and human) detected by flow cytometry. (b) Multilineage reconstitution was shown as the percentage of HSPCs (hCD45+/hCD34+), T cells (hCD45+/hCD3+), B cells (hCD45+/hCD19+), myeloid cells (hCD45+/hCD33+), NK cells (hCD45+/hCD56+/hCD16+), and erythroid cells (hCD235a+) in bone marrow of engrafted mice 26 weeks after transplantation. (c) The percentage of HBB+ cells within the population of hCD235a+ cells in the bone marrow of engrafted mice 16 or 26 weeks after transplantation, which was detected by flow cytometry. (d) Western blot analysis of the α-globin and β-globin expression in the bone marrow of engrafted mice 26 weeks after transplantation, with β-actin served as an internal control to relative expression analysis. The β-globin/α-globin ratio normalized to the average ratio in the control group is shown below. The full uncropped blot images

were given in the Online Resource. (e) Statistics of the β-globin/α-globin ratio in the bone marrow of engrafted mice 26 weeks after transplantation, as in (d), normalized to the average ratio in the control group. (f) Statistics of the functional reparation frequency of *HBB* CD41-42 (-TCTT) mutation in the input HSPCs and the cells derived from the bone marrow of engrafted mice 26 weeks after transplantation, which was detected by NGS. Ctrl indicated the mice transplanted with untransfected HSPCs, while Edited indicated the mice transplanted with HSPCs transfected with Cas9:sgRNA_1 RNP and ssODNs as templates. The data represent the mean ± SD for 2 to 13 engrafted mice transplanted by unedited or edited homozygote *HBB* CD41-42 (-TCTT) patient-derived CD34+ cells. Each dot represented a single recipient mouse. **, $P < 0.01$, *, $P < 0.05$ (by unpaired *t* test).

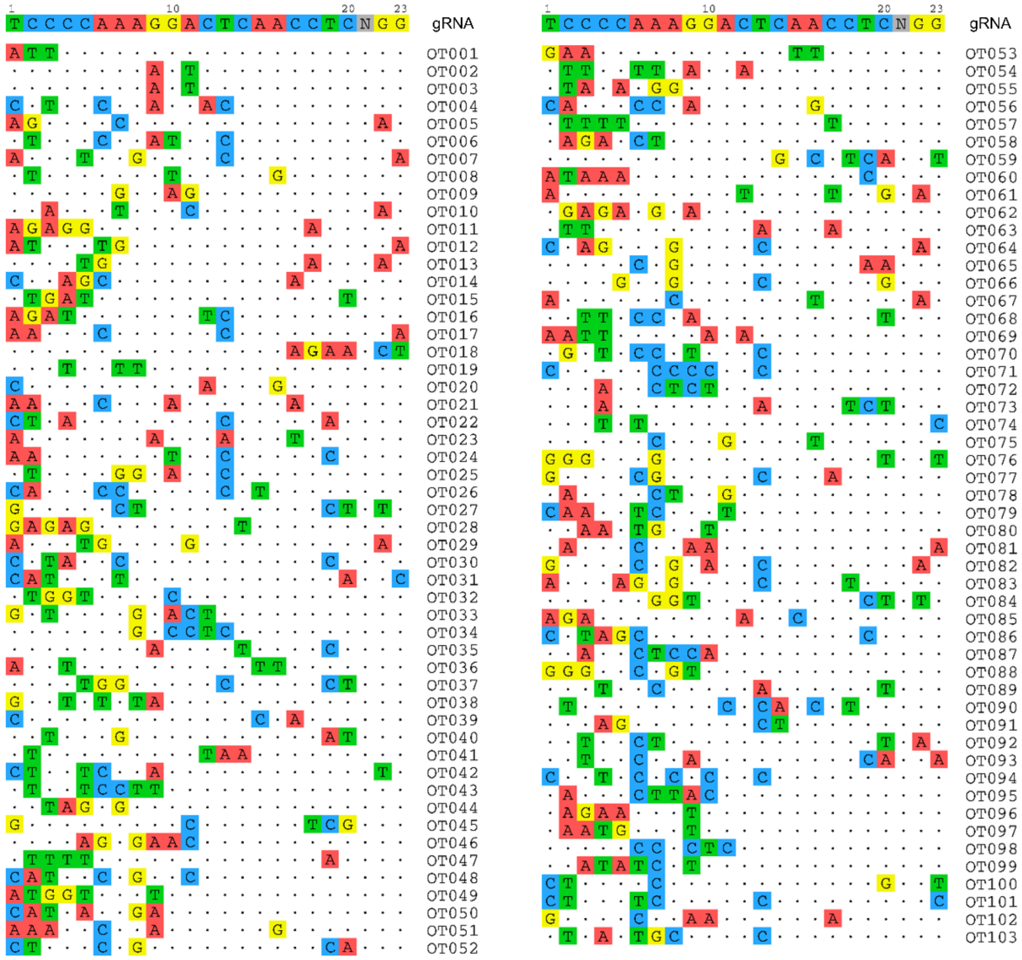

Fig. S7 Overview of the 103 candidate off-target sites for Cas9:sgRNA_1 RNP predicted by SITE-seq analysis and CRISPOR.

The candidate off-target sites prediction by both SITE-seq with 90 nmol RNP and either SITE-seq with 256 nmol RNP or CRISPOR was pointed out. The sequence of on-target sites and PAM was shown at the top. Dots represented matches to the on-target site, while the colored nucleotides represented mismatches.
